# Single vector platform vaccine protects against lethal respiratory challenge with Tier 1 select agents of anthrax, plague, and tularemia

**DOI:** 10.1038/s41598-018-24581-y

**Published:** 2018-05-03

**Authors:** Qingmei Jia, Richard Bowen, Barbara Jane Dillon, Saša Masleša-Galić, Brennan T. Chang, Austin C. Kaidi, Marcus A. Horwitz

**Affiliations:** 1 Division of Infectious Diseases, Department of Medicine, 37-121 Center for Health Sciences, School of Medicine, University of California – Los Angeles, 10833 Le Conte Avenue, Los Angeles, CA 90095-1688 USA; 2 Department of Biomedical Sciences, Colorado State University, Fort Collins, CO 80523 USA

## Abstract

*Bacillus anthracis*, *Yersinia pestis*, and *Francisella tularensis* are the causative agents of Tier 1 Select Agents anthrax, plague, and tularemia, respectively. Currently, there are no licensed vaccines against plague and tularemia and the licensed anthrax vaccine is suboptimal. Here we report *F. tularensis* LVS Δ*capB* (Live Vaccine Strain with a deletion in *capB*)*-* and attenuated multi-deletional *Listeria monocytogenes* (Lm)-vectored vaccines against all three aforementioned pathogens. We show that LVS Δ*capB*- and Lm-vectored vaccines express recombinant *B. anthracis*, *Y. pestis*, and *F. tularensis* immunoprotective antigens in broth and in macrophage-like cells and are non-toxic in mice. Homologous priming-boosting with the LVS Δ*capB*-vectored vaccines induces potent antigen-specific humoral and T-cell-mediated immune responses and potent protective immunity against lethal respiratory challenge with all three pathogens. Protection against anthrax was far superior to that obtained with the licensed AVA vaccine and protection against tularemia was comparable to or greater than that obtained with the toxic and unlicensed LVS vaccine. Heterologous priming-boosting with LVS Δ*capB*- and Lm-vectored *B. anthracis* and *Y. pestis* vaccines also induced potent protective immunity against lethal respiratory challenge with *B. anthracis* and *Y. pestis*. The single vaccine platform, especially the LVS Δ*capB*-vectored vaccine platform, can be extended readily to other pathogens.

## Introduction

*Bacillus anthracis*, *Yersinia pestis*, and *Francisella tularensis*, the causative agents of anthrax, plague, and tularemia, respectively, are classified as Tier 1 Select Agents of bioterrorism. When *B. anthracis*, *Y. pestis*, and *F. tularensis* infect humans by the respiratory route, the route of greatest concern in an intentional bioterrorist attack, they cause highly fatal diseases - pulmonary anthrax, pneumonic plague, and pneumonic tularemia, respectively. Pulmonary anthrax has a mortality as high as 100% untreated and 45% with treatment^1^; pneumonic plague is rapidly lethal (~50–90%) untreated^2^; and pneumonic tularemia has a mortality of up to 40–60% untreated and can be highly fatal even with appropriate antibiotic treatment^3^. Because *B. anthracis*, *Y. pestis* and *F. tularensis* are relatively easy to manufacture, environmentally hardy, and cause high mortality, they are considered among the most likely pathogens to be employed by terrorists and are consequently classified as Tier 1 Select Agents; indeed, they were developed as bioweapons during WWII and the Cold War^4,5^.

Although antibiotics can afford protection against *B. anthracis*, *Y. pestis* and *F. tularensis* in animal models, the critical period for treatment following aerosol challenge is very short (24–72 h)^6^. Furthermore, antibiotic-resistant strains of these pathogens can be developed by genetic engineering^4,7^, emerge from long-term antibiotic treatment^8^, or acquired naturally from transferable plasmids^9^. Hence, relying on currently available antibiotics to counter an intentional outbreak of anthrax, plague, or tularemia is not a practical public health plan. In view of the potential catastrophic consequences of the intentional airborne spread of these pathogens and the increasing development of antibiotic-resistant strains, vaccines are needed to protect against inhaled *B. anthracis*, *Y. pestis*, and *F. tularensis* and other Tier 1 Select Agents. The currently available licensed human anthrax vaccines are the U.S. anthrax vaccine adsorbed (AVA) and the U.K. anthrax vaccine precipitated (AVP); both are undefined acellular subunit vaccines, containing primarily the *B. anthracis* Protective Antigen (PA) with a lesser amount of Lethal Factor (LF) and other proteins. AVA requires 5 vaccinations followed by annual boosters and its duration of efficacy is unknown. In addition, AVA causes adverse reactions such as local soreness, redness, itching and swelling at the site of injection. The complexity of the immunization schedule and adverse effects of AVA make it unattractive. There are currently no licensed vaccines against plague or tularemia. The *Y. pestis* EV76 strain was developed and used in humans in the former Soviet Union; however, it has significant toxicity and is not licensed in the U.S.^2^. The *F. tularensis* Live Vaccine Strain (LVS) has been extensively studied in the U.S.; this unlicensed vaccine is relatively toxic and provides incomplete protection against aerosolized *F. tularensis*^10^. Practically speaking, a single vector platform vaccine against *F. tularensis*, *B. anthracis*, and *Y. pestis* that is highly efficacious is needed as it would simplify manufacture, regulatory approval, clinical evaluation, and vaccine administration, be more acceptable to people than multiple individual vaccines, and be less costly. Currently, no single vector platform vaccine against Tier 1 Select Agents is available.

In the case of *B. anthracis* and *Y. pestis*, subunit vaccines including *B. anthracis* PA and LF antigens or *Y. pestis* F1 capsular antigen and low calcium response V (LcrV) antigen in adjuvants or vaccines comprising a live attenuated heterologous vector expressing these antigens have induced strong protection in preclinical studies^11–14^. However, in the case of *F. tularensis*, subunit vaccines or vaccines comprising live attenuated heterologous vectors expressing *F. tularensis* proteins show relatively poor efficacy against high dose *F. tularensis* aerosol challenge in comparison with LVS, which itself is suboptimal^15,16^. Thus, protection against aerosolized highly virulent Type A *F. tularensis* strains requires a live homologous vector such as LVS or deletional mutants of LVS or Type A *F. tularensis* strains. Our live homologous LVS Δ*capB* vector has significant advantages over the alternative approaches of single-deletional (unsafe) or double-deletional (ineffective) mutants of virulent Type A *F. tularensis* in terms of safety, efficacy, and regulatory approval. While >10,000-fold less virulent than the toxic LVS strain in mice, LVS Δ*capB* is highly protective - ~100% protection against aerosolized *F. tularensis* SchuS4 after intranasal (i.n.) immunization, and strong protection after intradermal (i.d.) immunization^17^. rLVS Δ*capB* expressing *F. tularensis* proteins induces strong cellular and humoral immune responses and protection comparable to immunization with LVS after single i.n. and i.d. immunization either as a standalone vaccine or as a prime vaccine to animals heterologously boosted with recombinant *Listeria monocytogenes* (Lm) expressing IglC (rLm/iglC)^18,19^. However, whether multiple i.d. doses of the non-toxic rLVS Δ*capB* expressing the *F. tularensis* fusion protein comprising the immunodominant domains of IglA, IglB, and IglC (rLVS Δ*capB*/*iglABC*) would yield even greater protection, e.g. protection greater than that of LVS, has not been investigated. Finally, optimal immunoprotection against *Y. pestis* requires T-cell responses^20,21^ and the live vectors proposed here induce such responses.

Attenuated Lm vectors, including Lm Δ*actA*, Lm Δ*actA* Δ*inlB*, and Lm Δ*actA* Δ*inlB* ΔuvrAB *prfA*(G155S) (abbreviated as Lm Δ*actA* Δ*inlB prfA* hereafter), have been developed as vaccine vectors for delivery of cancer and infectious diseases antigens and have major advantages over other vectors as described by us and others^15,18,22–24^.

Here we describe a novel single vector vaccine platform against Tier I Select Agents *B. anthracis*, *Y. pestis*, and *F. tularensis* comprising LVS Δ*capB* as a vaccine vector to express heterologously immunoprotective domains of *B. anthracis* LF and PA antigens or *Y. pestis* F1 and LcrV antigens, or to overexpress homologously *F. tularensis* immunodominant domains of IglA, IglB, and IglC^19^, respectively. We compare the immunogenicity and efficacy of homologous priming-boosting with the single LVS Δ*capB* vectored vaccine platform with that of heterologous priming-boosting with the LVS Δ*capB* - Lm vectored vaccine platform where the vaccines express the same immunoprotective antigens. We show that homologous priming-boosting with individual rLVS Δ*capB* vaccines expressing *B. anthracis*, *Y. pestis*, and *F. tularensis* antigens induces potent protection against respiratory challenge with lethal doses of all three pathogens – potency superior to that of the existing licensed AVA vaccine against *B. anthracis* challenge and the unlicensed and toxic LVS vaccine against *F. tularensis* challenge.

## Results

### Construction and characterization of attenuated recombinant *B. anthracis* and *Y. pestis* vaccines

We constructed the following *B. anthracis* and *Y. pestis* vaccine candidates by using LVS Δ*capB*^17^ and Lm Δ*actA* Δ*inlB prfA*^25^ as vaccine vectors (Table S1): (1) rLVS Δ*capB*/Ba expressing the shuttle plasmid-encoded fusion protein of *B. anthracis* antigens LF amino-terminal PA binding domain [LFn, 255 aa]^26,27^ and the PA extended carboxy-terminal host cell receptor-binding domain [PAc, 183 aa]^12^ separated by a flexible linker GGSG (LFnPAc) and driven by the *F. tularensis bfr* promoter (upstream of FTT_1441)^28^; (2) rLVS Δ*capB*/Yp expressing the fusion protein of *Y. pestis* F1 and LcrV (F1V), separated by the GGSG linker and driven by the *F. novicida omp* promoter (upstream of FTN_1451)^29,30^; (3) rLm Δ*actA* Δ*inlB prfA*/ActAN-Ba and rLm Δ*actA* Δ*inlB prfA*/LLOss-Ba expressing the secreted forms of the fusion protein of *B. anthracis* LFnPAc downstream of the Lm *actA* promoter, ligated in frame with the *actA*-encoded N-terminal 100 amino acid of ActA (ActAN-BaLFnPAc), or downstream of the Lm *hly* promoter, ligated in frame with the *hly*-encoded Listeriolycin O (LLO) signal sequence (LLOss-BaLFnPAc)^24^; and (4) rLm Δ*actA* Δ*inlB prfA*/ActAN-Yp and rLm Δ*actA* Δ*inlB prfA*/LLOss-Yp expressing secreted forms of ActAN-YpF1V and LLOss-YpF1V, respectively.

**Figure 1:**
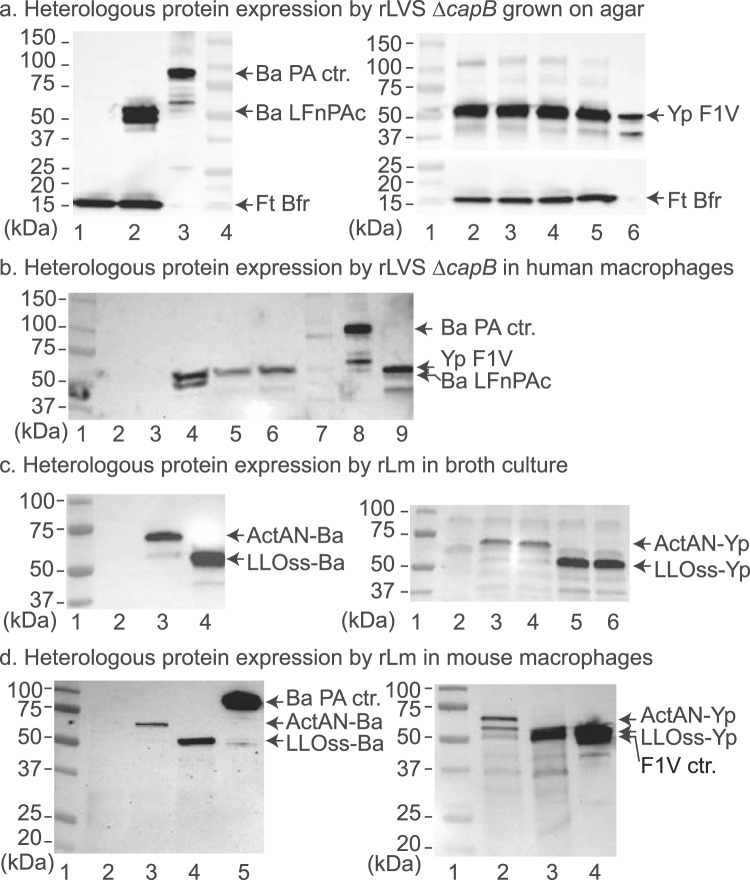
Expression of heterologous fusion proteins of *B. anthracis* and *Y. pestis* by rLVS Δ*capB* and rLm Δ*actA* Δ*inlB prfA* vaccines grown on agar and in infected macrophage-like cells. (**a**) Expression of *B. anthracis* (left) and *Y. pestis* (right) fusion proteins by rLVS Δ*capB* grown on agar. Single colonies of chocolate agar grown rLVS Δ*capB/*Ba and rLVS Δ*capB/*Yp (4 clones) were lysed in SDS sample buffer and lysates analyzed by Western blotting using a mixture of antibody to *B. anthracis* PA and to *F. tularensis* Bfr (left panel) or antibody to *Y. pestis* LcrV protein followed by antibody to Bfr (right panel). Left panel, lane 1, LVS Δ*capB* vector; lane 2, rLVS Δ*capB/*Ba; lane 3, PA protein control; lane 4, protein mass standards. Right panel, lane 1, protein mass standards; lanes 2–5, rLVS Δ*capB/*Yp; lane 6, monomer of F1-LcrV (F1V) protein control. (**b**) Expression of fusion proteins by rLVS Δ*capB* in infected human macrophage-like cells. Monocytic THP-1 cells seeded on 24-well plates and differentiated in the presence of PMA were left uninfected or infected with LVS Δ*capB*, rLVS Δ*capB*/Ba or rLVS Δ*capB*/Yp; cells were lysed at 24 h post infection, and cell lysates analyzed by Western blotting using a mixture of antibody to *B. anthracis* PA and to *Y. pestis* LcrV. Lanes 1 & 7, two different protein standards; lane 2, uninfected control; lane 3, LVS Δ*capB;* lane 4, rLVS Δ*capB*/Ba; lanes 5 and 6, two clones of rLVS Δ*capB/*Yp vaccines; lane 8, *B. anthracis* PA and degraded proteins; lane 9, *Y. pestis* F1-LcrV monomer protein and degraded proteins. (**c**) Expression and secretion of heterologous fusion proteins by rLm vaccines in broth. Culture filtrates of Lm vector or rLm vaccines were analyzed by Western blotting using antibody to *B. anthracis* PA (left panel) or to *Y. pestis* LcrV (right panel). Left panel, lane 1, protein mass standards; lane 2, Lm vector; lane 3, rLm/ActAN-Ba; lane 4, rLm/LLOss-Ba. Right panel, lane 1, protein mass standards; lane 2, Lm vector; lanes 3 & 4, two clones of rLm/ActAN-Yp; lanes 5 & 6, two clones of rLm/LLOss-Yp. (**d**) Expression of heterologous fusion proteins by rLm vaccines in infected mouse macrophage-like cells. Monolayers of J774A.1 cells were not infected or infected with a stationary culture of rLm vaccines similarly as described above in the legend to b . Lysates were subjected to Western blotting analysis using antibody to *B. anthracis* PA (left) or to *Y. pestis* LcrV (right). Left panel, lane 1, protein standards; lane 2, uninfected control; lane 3, rLm*/*ActAN-Ba; lane 4, rLm*/*LLOss-Ba; lane 5, PA protein. Right panel, lane 1, protein standards; lane 2, rLm*/*ActAN-Yp; lane 3, rLm*/*LLOss-Yp; lane 4, F1-LcrV protein control. (**a**–**d**) On the left border of each panel are listed the masses of protein standards; on the right border are listed the proteins of interest. Each blot was processed by using the Bio-Rad imaging system (ChemiDoc XRS) and Quantity One software, which allows the overlap of a white-light image, for visualization of the protein standards (**a**, left panel lane 4 and right panel lane 1; **b**–**d**, lane 1), and a chemiluminescent image, for visualization of the antibody-labeled protein bands. The full-length blots in panels b–d are shown in the Supplementary Information (Fig. S1).

Evaluation of the LVS Δ*capB*-vectored vaccines showed that the 51 kDa fusion protein of *B. anthracis*, LFnPAc, is expressed by rLVS Δ*capB*/Ba grown on agar (Fig. 1a, left panel, lane 2), but not by the LVS Δ*capB* vector control (lane 1), as detected by monoclonal antibody to *B. anthracis* PA protein, which also detected the 83-kDa PA protein (lane 3). Similarly, the 55 kDa fusion protein of *Y. pestis* F1V is expressed by rLVS Δ*capB*/Yp grown on agar (Fig. 1a, right panel, lanes 2–5), as detected by goat polyclonal antibody to *Y. pestis* LcrV, and has a molecular mass similar to the F1-LcrV monomer protein (lane 6). *F. tularensis* Bfr was detected as a 17-kDa protein^31^ from the LVS Δ*capB* vector as well as from rLVS Δ*capB*/Ba (Fig. 1a, left panel, lanes 1 and 2) and rLVS Δ*capB*/Yp (Fig. 1a, right panel, lanes 2–5). The expression of the fusion proteins of *B. anthracis* and *Y. pestis* was also detected in human macrophage-like THP-1 cells infected with rLVS Δ*capB*/Ba as double bands (Fig. 1b, lane 4) and rLVS Δ*capB*/Yp as a single band (Fig. 1b, lanes 5 & 6), respectively. Although the rLVS Δ*capB* vaccine strains showed somewhat delayed growth kinetics in broth (Fig. S2a), they grew similarly to the parental LVS Δ*capB* strain in infected THP-1 cells (data not shown) and in mouse macrophage-like J774A.1 cells (Fig. S3a).

Lm-vectored vaccines were assessed for expression and secretion of *B. anthracis* and *Y. pestis* fusion proteins after growth in broth and in infected mouse macrophage-live J774A.1 cells by evaluating culture filtrates by Western blotting using antibodies specific to *B. anthracis* PA or to *Y. pestis* LcrV. As shown in Fig. 1c, the antibody to PA or LcrV detected major protein bands of 59-kDa ActAN-BaLFnPAc (ActAN-Ba) (Fig. 1c, left panel, lane 3), 51-kDa LLOss-BaLFnPAc (LLOss-Ba) (lane 4), 63-kDa ActAN-YpF1V (ActAN-Yp) (Fig. 1c, right panel, lanes 3 & 4), and 56-kDa LLOss-YpF1V (LLOss-Yp) (lanes 5 & 6), expressed and secreted by rLm Δ*actA* Δ*inlB prfA*/ActAN-Ba, rLm Δ*actA* Δ*inlB prfA*/LLOss-Ba, rLm Δ*actA* Δ*inlB prfA*/ActAN-Yp, and rLm Δ*actA* Δ*inlB prfA*/LLOss-Yp, respectively, as predicted, but not by the Lm vector controls (Fig. 1c, left and right panels, lane 2). The expression of the fusion proteins of *B. anthracis* and *Y. pestis* was also detected from J774A.1 cells infected with rLm Δ*actA* Δ*inlB prfA*/ActAN-Ba (Fig. 1d, left panel, lane 3) or rLm Δ*actA* Δ*inlB prfA*/LLOss-Ba (lane 4) or with rLm Δ*actA* Δ*inlB prfA*/ActAN-Yp (Fig. 1d, right panel, lane 2) or rLm Δ*actA* Δ*inlB prfA*/LLOss-Yp (lane 3), respectively. The rLm vaccines strains grew similarly to the parental Lm Δ*actA* Δ*inlB prfA* strain in broth (Fig. S2b) and in infected J774A.1 cells (Fig. S3b). Because rLm Δ*actA* Δ*inlB prfA*/LLOss-Ba and rLm Δ*actA* Δ*inlB prfA*/LLOss-Yp expressed the *B. anthracis* and *Y. pestis* fusion proteins more abundantly in both broth and in macrophages than rLm Δ*actA* Δ*inlB prfA*/ActAN-Ba and rLm Δ*actA* Δ*inlB prfA*/ActAN-Yp vaccines, respectively, we chose to use rLm Δ*actA* Δ*inlB prfA*/LLOss-Ba and rLm Δ*actA* Δ*inlB prfA*/LLOss-Yp in the following studies where they are hereafter referred to as rLm/Ba and rLm/Yp, respectively.

Initial *in vivo* studies examined dissemination, clearance, and plasmid stability of the newly constructed *B. anthracis* and *Y. pestis* vaccines. Our results showed that rLVS Δ*capB*/Ba grew and disseminated similarly to the parental LVS Δ*capB*, while rLVS Δ*capB*/Yp showed delayed growth and dissemination (Figs S4 and S5). The shuttle plasmid-encoded antigen expression cassettes in rLVS Δ*capB*/Ba and rLVS Δ*capB*/Yp were stably maintained in mouse liver, spleen, local skin (after i.d. administration), and lung (after i.n. administration) up to 14 days post vaccination (data not shown); The rLm/Ba and rLm/Yp vaccines also showed systemic dissemination, similar to the parental Lm vector, and all were cleared by Day 7 post vaccination (Fig. S6).

### Vaccine immunogenicity and protective immunity against pulmonary anthrax

**Figure 2:**
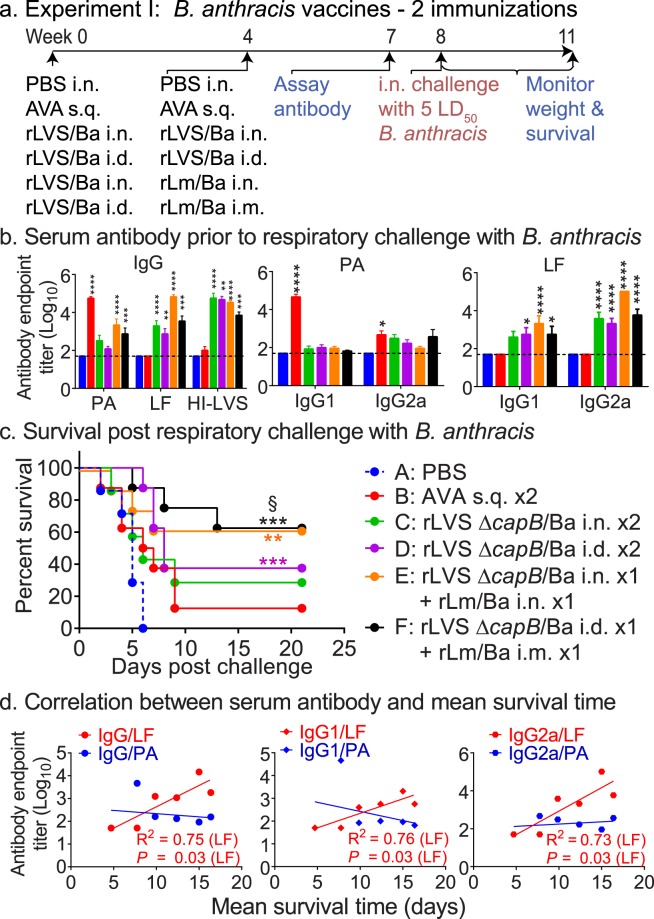
Two immunizations by homologous priming-boosting with rLVS Δ*capB*/Ba or by heterologous priming-boosting with rLVS Δ*capB*/Ba - rLm Δ*actA* Δ*inlB prfA*/Ba induce humoral immune responses and protective immunity against respiratory challenge with virulent *B. anthracis* Ames spores. (**a**) Experiment schedule. Mice (n = 8/group) were immunized homologously twice, 4 weeks apart, with PBS intranasally (i.n.) (Sham), AVA (0.025 ml) subcutaneously (s.q.), or 10^6^ CFU rLVS Δ*capB*/Ba (rLVS/Ba) i.n. or intradermally (i.d.), or heterologously with 10^6^ CFU rLVS Δ*capB/*Ba i.n. or i.d. and rLm/Ba i.n. or intramuscularly (i.m.), 4 weeks apart; bled at Week 7; challenged at week 8 with *B. anthracis* Ames spores (205,000/mouse, ~5 LD_50_); and monitored for survival for three weeks, as indicated. (**b**) Serum antibody after vaccination. Sera were assayed for antibody endpoint titer to *B. anthracis* PA and LF antigens and to *F. tularensis* heat-inactivated LVS (HI-LVS). Values are mean + SEM of serum antibody endpoint titer for n = 8 mice per group. Differences among individual groups were evaluated by Two-way ANOVA with Tukey’s correction. Values significantly different from the Sham group are marked with asterisks over the comparison groups. *P < 0.05; **P < 0.01; ***P < 0.001; ****P < 0.0001. (**c**) Survival after vaccination and challenge. The survival curve of each vaccinated group is compared with that of Group A (Sham) or Group B (AVA) by the log-rank test (Mantel-cox); *P* values for individual vaccine groups significantly different from the Sham or AVA group are marked with asterisks and “§”, respectively, color-coded to the color of the vaccine symbol; **P < 0.01; ***P < 0.001 vs. Sham group; ^§^*P* < 0.05 vs. AVA group. (**d**) Correlation between serum antibody and mean survival time. Linear regression was used to obtain values for the slope and intercept and the correlation coefficient (R^2^) between pre-challenge serum antibody and post-challenge mean survival time at 21 days post-challenge. Two-tailed *P* values were calculated for the correlation.

To examine the protective immunity of the LVS Δ*capB-* and Lm-vectored *B. anthracis* vaccines, we sham-immunized the mice, or immunized them twice with AVA (BEI NR-2642, BioThrax) subcutaneously (s.q., its standard route of immunization) in a 100-μL volume after the AVA vaccine was diluted (0.25 ml: 0.75 ml) in sterile PBS^32^, or immunized homologously with rLVS Δ*capB*/Ba mucosally (i.n.) or systemically (i.d.), or heterologously primed with rLVS Δ*capB*/Ba (i.n. or i.d.) and subsequently boosted with rLm/Ba [i.n. or i.m.; i.m. is the most immunogenic systemic route for Lm^33^], as indicated in Fig. 2a. All mice were bled at Week 7, one week prior to challenge; challenged at Week 8 with 205,000 *B. anthracis* Ames spores (~5 LD_50_; the pre-determined LD_50_ for *B. anthracis* Ames spores was ~40,000 CFU); and monitored for survival for three weeks. Homologous priming-boosting with rLVS Δ*capB*/Ba i.n. or i.d. and heterologous priming-boosting with rLVS Δ*capB*/Ba – rLm/Ba, i.n./i.n. or i.d./i.m induced elevated PA-, LF- and HI-LVS-specific serum antibodies (Fig. 2b, leftmost panel); the LF- and HI-LVS antibody titers were significantly higher than those of sham- and AVA-immunized mice (Fig. 2b, leftmost and rightmost panels); these live-vectored vaccines tended to induce more LF- and PA-specific IgG2a than IgG1 antibody, representing stimulation of Th1 and Th2 immune responses, respectively (Fig. 2b, middle and rightmost panels). AVA vaccination induced high antibody titers, dominated by IgG1, specific to PA, the major component of AVA vaccine, but not to LF and HI-LVS (Fig. 2b). Systemic homologous priming-boosting with rLVS Δ*capB*/Ba (Group D) and heterologous priming-boosting with rLVS Δ*capB*/Ba – rLm/Ba i.n./i.n. (Group E) and i.d./i.m (Group F) showed significant protection (*P* < 0.001–0.01 vs. sham-immunized mice) (Fig. 2c). The licensed AVA vaccine did not induce significant protection compared with sham-immunized mice, and heterologous priming-boosting with rLVS Δ*capB*/Ba – rLm/Ba systemically was significantly more protective than the AVA vaccine (*P* = 0.02) (Fig. 2c). Interestingly, mean survival time three weeks post-challenge correlated with pre-challenge serum antibody to LF but not to PA (Fig. 2d).

**Figure 3:**
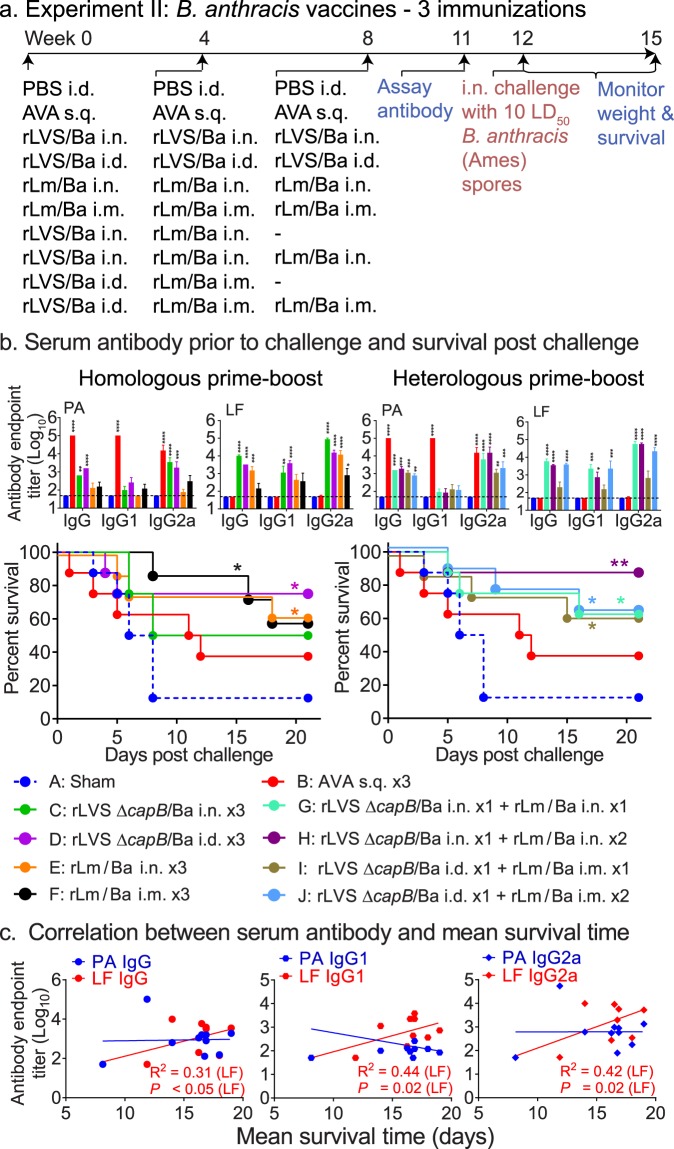
Three immunizations by homologous priming-boosting with rLVS Δ*capB*/Ba or by heterologous priming-boosting with rLVS Δ*capB*/Ba – rLm Δ*actA* Δ*inlB prfA*/Ba induce high-level antibody responses and potent protective immunity against virulent *B. anthracis* respiratory challenge. (**a**) Experiment schedule. Mice (n = 8/group) were immunized two or three times homologously with 10^6^ CFU rLVS Δ*capB*/Ba (rLVS/Ba) or rLm Δ*actA* Δ*inlB prfA*/Ba (rLm/Ba) or heterologously first with rLVS/Ba and subsequently with rLm/Ba, as indicated. Controls were sham-immunized with PBS i.d. or with AVA s.q. three times. All mice were bled at week 11; challenged at week 12 with *B. anthracis* Ames spores (371,000 CFU, ~10 LD_50_); and monitored for survival for 3 weeks post-challenge. (**b**) Serum antibody prior to challenge and survival post challenge. Top panels. Sera were assayed for IgG or IgG subtypes IgG1 and IgG2a to *B. anthracis* PA and LF proteins, as indicated, after homologous (left two panels) and heterologous (right two panels) prime-boost vaccination. Values are mean + SEM of serum antibody endpoint titer for n = 8 per group. Differences in serum endpoint titer among individual groups were analyzed by two-way ANOVA with Tukey’s corrections. *P < 0.05; **P < 0.01; ****P < 0.0001 vs. Sham group. Bottom panels. The survival curve of each vaccinated group, color-coded as indicated in panel b, after homologous (left) and heterologous (right) prime-boost vaccination and challenge is compared with that of the Sham group by the log-rank test (Mantel-cox); *P* values for vaccine groups that are significantly different from the Sham group are marked with one or more asterisks color-coded to the color of the vaccine symbol. *P < 0.05 and **P < 0.01. (**c**) Correlation between serum antibody and mean survival time. The correlation coefficient (R^2^) and one-tailed *P* values were obtained as described in legend to Fig. 2d.

Subsequently, we explored the efficacy of three homologous (rLVS Δ*capB*/Ba or rLm/Ba) or heterologous (one rLVS Δ*capB*/Ba prime + two rLm/Ba boosts) immunizations, both mucosally (i.n.) and systemically (i.d. for rLVS Δ*capB*/Ba and i.m. for rLm/Ba), and compared them with that of sham or AVA immunization (s.q.), and with one rLVS Δ*capB*/Ba prime + one rLm/Ba boost vaccination, as depicted in Fig. 3a. The immunized animals were bled, subsequently challenged with 371,000 *B. anthracis* Ames spores (~10 LD_50_), and monitored for 3 weeks (Fig. 3a). As shown on the left side of Fig. 3b, mice homologously primed-boosted with rLVS Δ*capB*/Ba i.n. (Group C) or i.d. (Group D) or with rLm/Ba i.n. (Group E) or i.m. (Groups F) produced significantly greater amounts of *B. anthracis* PA and/or LF antigen-specific serum IgG antibody, dominated by subtype IgG2a, than sham-immunized mice (Fig. 3b, top left two panels), consistent with the results from Experiment I (Fig. 2a and b) and additional experiments (Fig. S7a–d, Group C). Of note, as in the previous experiment, mice immunized with AVA produced PA-specific antibody, but did not produce LF-specific serum antibody. Upon challenge, mice homologously primed-boosted with rLVS Δ*capB*/Ba or rLm/Ba, systemically or mucosally, had greater survival than sham- and AVA-immunized mice; the survival of mice immunized systemically with rLVS Δ*capB*/Ba and rLm/Ba and mucosally with rLm/Ba was significantly greater than that of the sham-immunized mice (Fig. 3b, bottom left panel).

As shown on the right side of Fig. 3b, mice heterologously primed-boosted with rLVS Δ*capB*/Ba - rLm/Ba, mucosally (i.n./i.n.) (Group G & H) or systemically (i.d./i.m.) (Group I & J) also produced significantly greater amounts of *B. anthracis* PA and LF antigen-specific serum IgG antibody than sham-immunized mice, dominated by subtype IgG2a to PA and LF (Fig. 3b, top right two panels; Fig. S7b–e, Groups E and F), and elevated levels of serum antibodies that neutralized anthrax toxin (assayed in mouse macrophage cell line J774A.1), as did AVA-immunized mice (Fig. S7f, Groups B, E, and F). After challenge, these heterologously primed-boosted mice (Groups G, H, I, J) showed significantly increased survival compared with the sham-immunized mice (*P* < 0.05 or *P* < 0.01), whether boosted only once (Groups G and I) or twice (Groups H and J) with rLm/Ba (Fig. 3b, bottom, right panel). In contrast, survival of mice immunized with the AVA vaccine was not significantly different from that of sham-immunized mice (*P* = 0.3) (Fig. 3b, bottom panels). These results indicate that both systemic and mucosal homologous priming-boosting with rLVS Δ*capB*/Ba or rLm/Ba and both systemic and mucosal heterologous priming-boosting with rLVS Δ*capB*/Ba - rLm/Ba induce strong protective immunity against lethal respiratory challenge with *B. anthracis* spores. As in the previous challenge experiment, mean survival time 3 weeks post-challenge correlated with pre-challenge serum antibody to LF but not to PA (Fig. 3c) or to toxin neutralizing antibody (Fig. S8).

**Figure 4:**
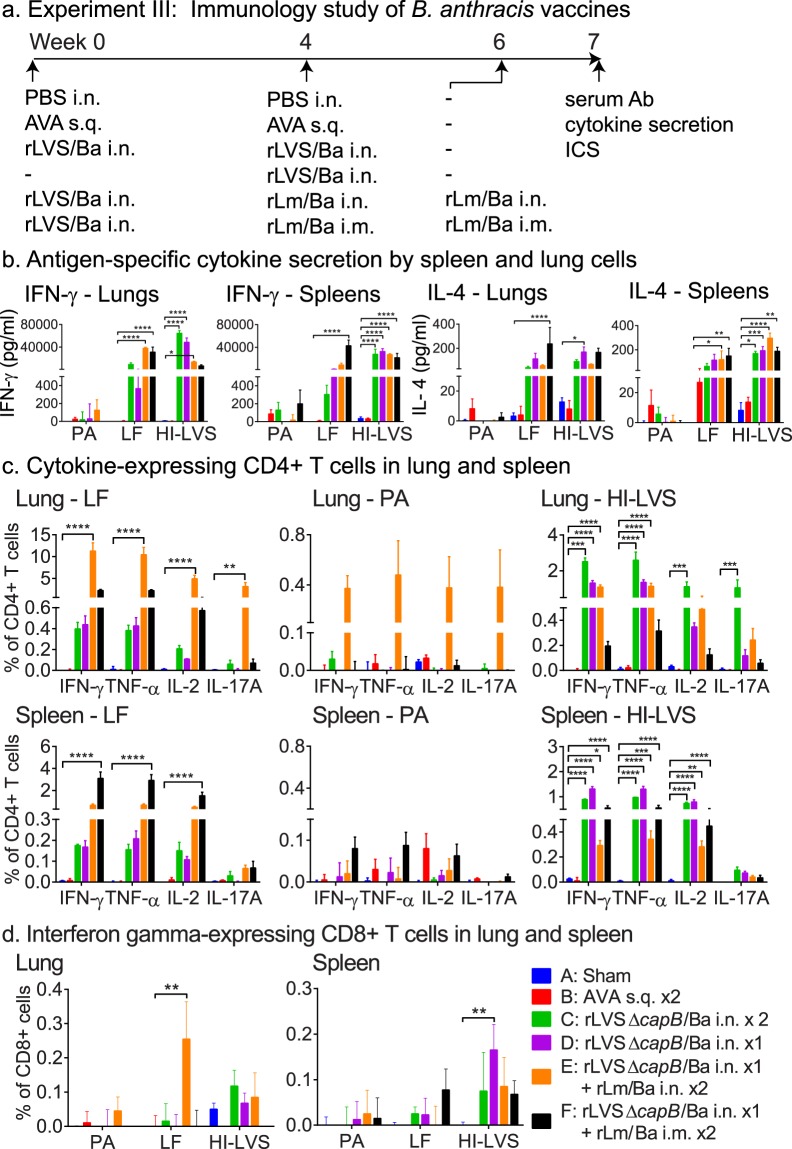
Homologous prime-boost vaccination with rLVS Δ*capB*/Ba or heterologous prime-boost vaccination with rLVS Δ*capB*/Ba - rLm Δ*actA* Δ*inlB prfA*/Ba induces antigen-specific cell-mediated immune responses. (**a**) Experiment schedule. Mice (n = 4/group) were immunized homologously with PBS i.n. (Sham), AVA s.q., or 10^6^ CFU rLVS Δ*capB*/Ba (rLVS/Ba) i.n., or heterologously with rLVS/Ba i.n. followed by 10^6^ CFU rLm Δ*actA* Δ*inlB prfA*/Ba (rLm/Ba) i.n. or i.m. as indicated. At week 7, all mice were bled, euthanized, and their lung and spleen cells assayed for cytokine secretion and intracellular cytokine staining. (**b**) Antigen-specific cytokine secretion. Single cell suspensions of lung and spleen cells were stimulated with *B. anthracis* PA or LF proteins or *F. tularensis* heat-inactivated LVS (HI-LVS) for 3 days, as indicated, and cell supernatants assayed for interferon gamma (IFN-γ) (left two panels) and IL-4 (right two panels) by ELISA. Shown are the amounts of IFN-γ and IL-4 in the culture supernate in response to PA, LF, and HI-LVS. (**c**,**d**) Cytokine-expressing CD4+ (c) and CD8+ (d) T cells. Lung and spleen cells were stimulated with LF, PA, or HI-LVS as indicated at the top of the panels (c) or on the horizontal axis (d) and assayed by intracellular cytokine staining for CD4+ (c) and CD8+ (d) T cells expressing IFN-γ, TNF-α, IL-2, and/or IL-17A. Shown are the frequencies of CD4+ T cells expressing IFN-γ, TNF-α, IL-2, and/or IL-17A (c) and CD8+ T cells expressing IFN-γ (d). Values in **b**–**d** are means + SEM. Differences among individual groups were evaluated by Two-way ANOVA with Tukey’s correction. Values significantly different from the Sham group are marked with asterisk(s) over brackets above the comparison groups; *P < 0.05; **P < 0.01; ***P < 0.001; ****P < 0.0001. Results shown are representative of three similar experiments.

**Figure 5:**
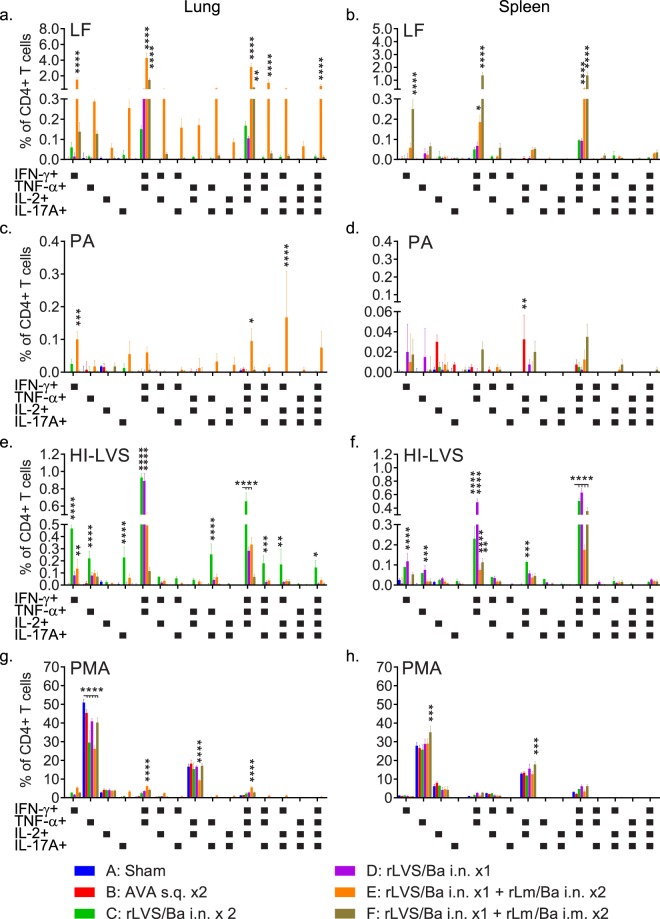
Heterologous prime-boost vaccination with rLVS Δ*capB*/Ba - rLm Δ*actA* Δ*inlB prfA*/Ba induces elevated frequencies of LF- and PA-specific polyfunctional lung and spleen CD4+ T-cells producing IFN-γ, IL-2, TNF-α, and IL-17. As described in Fig. 4a and indicated by the colors and letters at the bottom of the figure, mice (n = 4/group) were sham-immunized or immunized with various vaccines. Lung (left panels) and spleen (right panels) cells were stimulated with LF (**a**,**b**), PA (**c**,**d**), HI-LVS (**e**,**f**), or PMA (**g**,**h**) and assayed by intracellular cytokine staining for 15 possible combinations of CD4+ T-cells expressing IFN-γ, TNF-α, IL-2, and/or IL-17A. Values are means + SEM and differences among individual groups were analyzed by Two-way ANOVA with Tukey’s multiple comparisons test (Prism). Values significantly different from the Sham group are marked with asterisks over the comparison groups. In panels e, f and g, the Sham group is indicated by a short bar with an open end and the comparison groups are indicated by a short vertical line across the short bar. *P < 0.05; **P < 0.01; ***P < 0.001; ****P < 0.0001. Results shown are representative of three similar experiments.

To investigate T-cell mediated immune responses induced by rLVS Δ*capB* and rLm vaccines, we immunized mice, observed them for signs of discomfort or weight loss, and assayed their lung and spleen cells for cytokine secretion and intracellular cytokine staining in response to *in vitro* stimulation with *B. anthracis* and *F. tularensis* antigens (Figs 4a and S9). After vaccination with rLVS Δ*capB*/Ba i.n., rLm/Ba i.n. or i.m., or AVA s.q., mice did not show signs of significant discomfort (e.g. ruffled fur) or weight loss (data not shown). As expected, mice immunized with the rLVS Δ*capB*/Ba and rLm/Ba anthrax vaccines developed T-cell mediated immune responses, dominated by Th1 responses (Figs 4 and 5). Lung and spleen cells of mice immunized with rLVS Δ*capB*/Ba i.n. twice (Group C) or once (Group D) or heterologously primed-boosted with rLVS Δ*capB/*Ba - rLm/Ba i.n./i.n. (Group E) or i.n./i.m. (Group F) secreted much higher levels of IFN-γ and IL-4 in response to LF and HI-LVS than sham- (statistically significant) or AVA-immunized mice (Fig. 4b). In response to LF and HI-LVS, these mice had elevated levels of lung and spleen CD4+ T cells expressing IFN-γ, TNF-α, IL-2, and IL-17A compared with sham- and AVA-immunized mice and greater frequencies of polyfunctional CD4+ T cells expressing IFN-γ, TNF-α, IL-2, and/or IL-17 than sham-immunized mice (Figs. 4c, 5); the heterologously immunized mice had especially high levels of cytokine-expressing CD4+ T cells (Fig. 4c, leftmost and rightmost upper and lower panels), and modestly increased levels of CD8+ T-cells secreting IFN-γ in the lung when boosted i.n. and in the spleen when boosted i.m. (Fig. 4d). In response to PA, CD4+ T-cells from mice heterologously primed-boosted by the i.n. route also had increased cytokine-producing cells in the lung and to a much less extent in the spleen; not unexpectedly, when the booster was instead administered systemically (i.m.), there were increased cytokine positive  CD4+ T-cells in the spleen (Fig. 4c, middle upper and lower panels). With a few minor exceptions, the AVA vaccine induced very poor cell-mediated immune responses (Fig. 4b–d). Mice immunized i.d. once with rLVS Δ*capB*/Ba or heterologously primed-boosted with rLVS Δ*capB*/Ba i.d. – rLm/Ba i.m. also showed enhanced IFN-γ secretion by lung and spleen cells and increased frequencies of lung and spleen CD4+ T cells expressing IFN-γ, TNF-α, IL-2 and/or IL-17A in response to LF and HI-LVS compared with sham-immunized mice and mice immunized with AVA (data not shown). These results indicate that both homologous priming-boosting with rLVS Δ*capB*/Ba and heterologous priming-boosting with rLVS Δ*capB*/Ba – rLm/Ba vaccines induce *F. tularensis* and *B. anthracis* antigen-specific Th1-type cytokine secretion and polyfunctional CD4+ T cells.

### Vaccine immunogenicity and protective immunity against pneumonic plague

**Figure 6:**
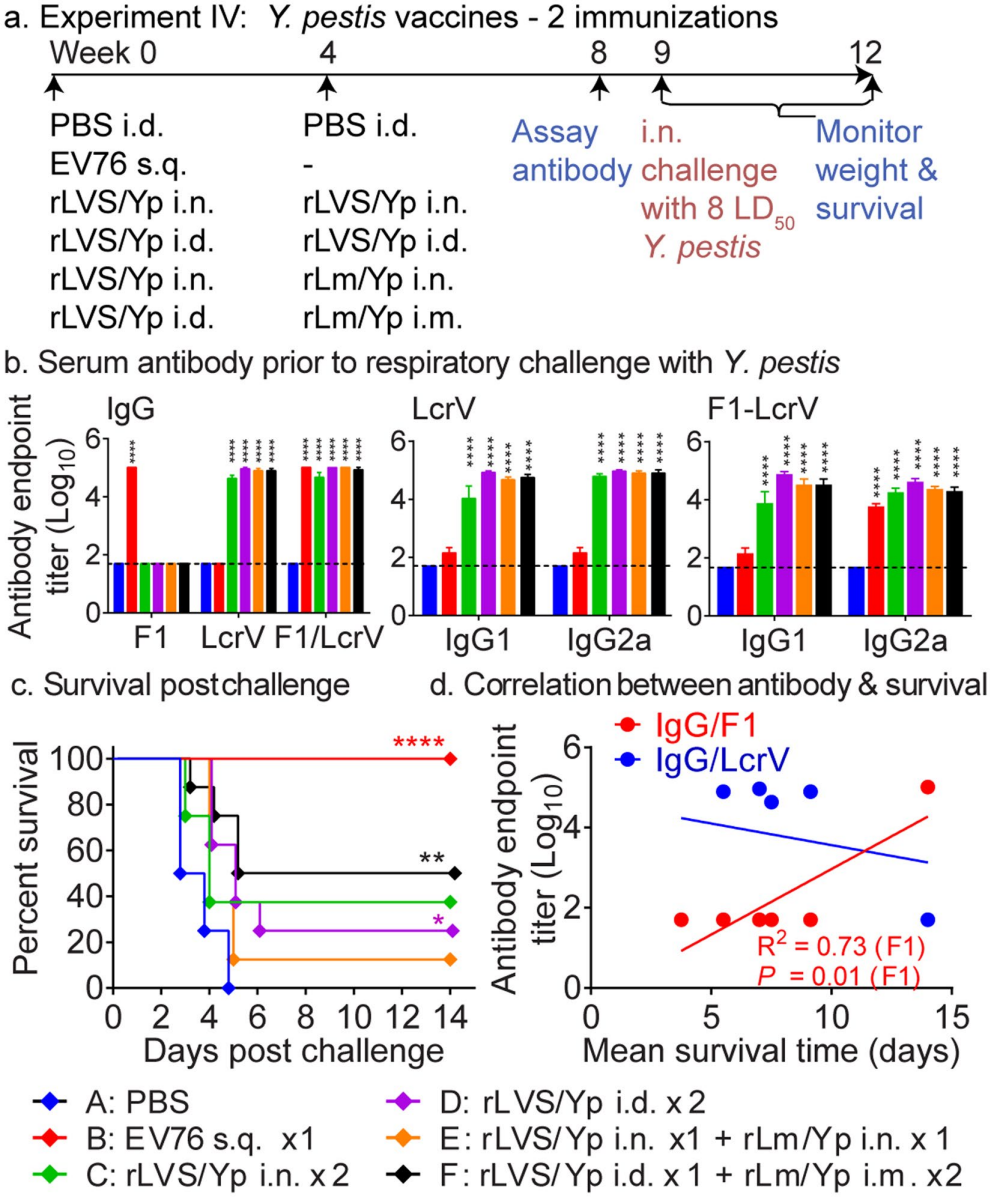
Two immunizations by homologous priming-boosting with rLVS Δ*capB*/Yp or heterologous priming-boosting with rLVS Δ*capB* - rLm Δ*actA* Δ*inlB prfA*/Yp induces humoral immune responses and protective immunity against respiratory challenge with virulent *Y. pestis*. (**a**) Experiment schedule. Mice (n = 8/group) were immunized homologously twice with PBS (Sham) or 10^6^ CFU LVS Δ*capB*/Yp (rLVS/Yp) or once with 10^6^ CFU EV76, or heterologously first with 10^6^ CFU rLVS Δ*capB/*Yp and then with 10^6^ CFU rLm Δ*actA* Δ*inlB prfA*/Yp (rLm/Yp). All mice were bled at Week 8; challenged at week 9 with virulent *Y. pestis* (CO92) (1,900 CFU/mouse, ~8 LD_50_); and monitored for survival for three weeks, as indicated. (**b**) Serum antibody after vaccination. Sera were assayed for IgG antibody specific to F1, LcrV, or the monomer of F1-LcrV proteins (leftmost panel) and subtypes IgG1 and IgG2a antibodies specific to LcrV or F1-LcrV (middle and rightmost panels, resp.). Data are mean + SEM of serum antibody endpoint titer for n = 8 per group. Differences among individual groups were analyzed by two-way ANOVA with Tukey’s correction. ****P < 0.0001 vs. Sham. (**c**) Survival after challenge. The survival curve of each vaccinated group is compared with the Sham group (Group A) by the log-rank test (Mantel-cox); *P* values that are significantly different from the Sham group are marked with one or more asterisks color-coded to the color of the vaccine symbol. There were no changes in percent survival of mice in any group after day 14 post challenge until the end of the experiment. *P < 0.05; **P < 0.01; ***P < 0.001; ****P < 0.0001. (**d**) Correlation between serum antibody and mean survival time. The correlation coefficient (R^2^) and one-tailed *P* values were obtained as described in legend to Fig. 2D.

To evaluate the protective efficacy of rLVS Δ*capB*- and rLm-vectored *Y. pestis* vaccines administered by the mucosal and systemic routes, we sham-immunized mice, or immunized them once with the unlicensed EV76 vaccine s.q. (its standard route of administration), or twice homologously with rLVS Δ*capB*/Yp i.n. or i.d. or heterologously primed-boosted with rLVS Δ*capB*/Yp (i.n. or i.d.) - rLm/Yp (i.n. or i.m., respectively), as indicated in Fig. 6a. All mice were bled at Week 8; challenged at Week 9 with 1900 CFU *Y. pestis* CO92 strain (~8 LD_50_; the pre-determined LD_50_ for *Y. pestis* CO92 was ~250 CFU), and monitored for signs of illness, weight change, and survival for three weeks. Mice immunized with rLVS Δ*capB*/Yp i.d or with rLm/Yp i.m. did not show signs of significant discomfort (e.g. ruffled fur) or weight loss (data not shown); in one experiment, mice immunized with rLVS Δ*capB*/Yp i.n. showed transient mild weight loss (~5%) but rapidly recovered such that their weights matched that of sham-immunized controls by at least Day 6 post–vaccination (data not shown). Homologous priming-boosting with rLVS Δ*capB*/Yp i.n. or i.d. (Groups C, D) or heterologous priming-boosting with rLVS Δ*capB*/Yp – rLm/Yp i.n. or i.m. (Groups E, F) induced significantly elevated serum antibody titers to LcrV protein and to F1/LcrV fusion protein, balanced between IgG1 and IgG2a, compared with sham-immunization (Fig. 6b) (Fig. S10a,b, upper panel, Groups B, E, & F). The majority of the IgG antibody induced by LVS Δ*capB*- and Lm-vectored vaccines was directed to LcrV. In contrast, mice immunized with EV76 produced very little antibody to LcrV (small amounts of IgG1 and IgG2a were evident) but substantial amounts of IgG to F1 and F1-LcrV (Fig. 6b).

Systemic homologous priming-boosting with rLVS Δ*capB*/Yp i.d. (Group D) or heterologous priming-boosting with rLVS Δ*capB*/Yp – rLm/Yp (i.d./i.m.) (Group F) showed significant protection against challenge with *Y. pestis* CO92 compared with sham-immunized mice (*P* = 0.02 and *P* = 0.01, respectively), albeit less than EV76 (Fig. 6c). Mean survival time three weeks post-challenge was correlated with pre-challenge serum IgG antibody to F1 but not to LcrV (Fig. 6d).

**Figure 7:**
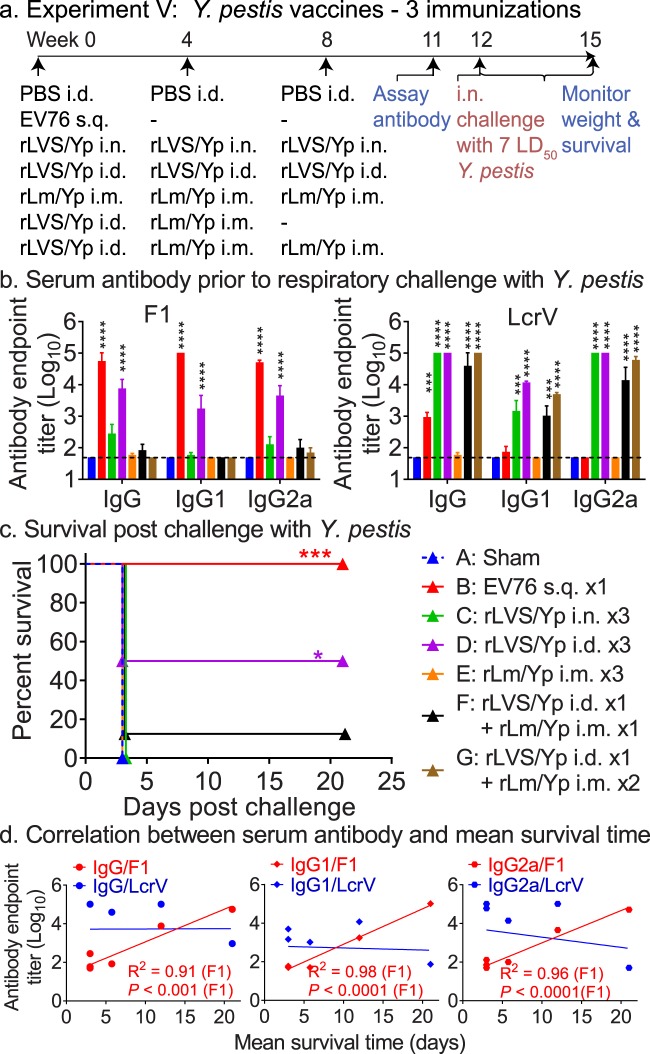
Three systemic immunizations by homologous priming-boosting with LVS Δ*capB*/Yp induce high level antibody responses and potent protective immunity against respiratory challenge with virulent *Y. pestis* CO92 strain. (**a**) Experiment schedule. Mice (n = 8/group) were immunized homologously three times with 10^6^ CFU rLVS Δ*capB*/Yp (rLVS/Yp) or with 10^6^ CFU rLm Δ*actA* Δ*inlB prfA*/Yp (rLm/Yp) or heterologously with rLVS Δ*capB*/Yp and subsequently rLm/Yp. Mice vaccinated with PBS i.d. (Sham, Group A) or 10^6^ CFU *Y. pestis* vaccine strain EV76 served as controls. All the mice were bled at Week 11; challenged with *Y. pestis* CO92 (1,800 CFU/mouse, ~7 LD_50_) at Week 12; and monitored for survival for 3 weeks post-challenge, as indicated. (**b**) Serum antibody after vaccination. Sera were assayed for antibody endpoint titer of IgG or IgG subtypes IgG1 and IgG2a to *Y. pestis* F1 (left panel) and LcrV (right panel) proteins. Data are mean + SEM of serum antibody endpoint titer for n = 8 per group. Differences among individual groups were analyzed by two-way ANOVA with Tukey’s correction. ***P < 0.001; ****P < 0.0001 vs. Sham. (**c**) Survival after vaccination and challenge. The survival curve of each vaccinated group is compared with that of the Sham group by the log-rank test (Mantel-cox); *P* values for vaccine groups significantly different from the Sham group are marked with one or more asterisks color-coded to the color of the vaccine symbol. *P < 0.05; ***P < 0.001. (**d**) Correlation between serum antibody and mean survival time. The correlation coefficient (R^2^) and one-tailed *P* values were obtained as described in legend to Fig. 2D.

Subsequently, we explored the efficacy of three homologous immunizations, both mucosally (i.n. for rLVS Δ*capB*/Yp) and systemically (i.d. for rLVS Δ*capB*/Yp and i.m. for rLm/Yp) and three heterologous immunizations systemically, as described in Fig. 7a. Controls were sham-immunized with PBS, administered the EV76 vaccine once s.q, or primed-boosted with rLVS Δ*capB*/Yp – rLm/Yp once systemically. Mice were bled; subsequently challenged with 1800 CFU virulent *Y. pestis* CO92 (~7 LD_50_); and monitored for survival for three weeks. Among the vaccines and administration regimens tested, three homologous priming-boosting immunizations with rLVS Δ*capB*/Yp i.d. (Group D) induced significantly greater serum antibody titers to F1 antigen than sham-immunized mice, balanced between subtypes IgG1 and IgG2a (Fig. 7b, left panel), similar to mice vaccinated with EV76. Homologous priming-boosting with rLVS Δ*capB*/Yp (i.n. or i.d) (Groups C, D) but not with rLm/Yp (i.m.) (Group E), and heterologous priming-boosting systemically (with one or two boosts) (Groups F, G) induced significantly elevated LcrV-specific IgG antibody compared with sham-immunized mice, balanced between subtypes IgG1 and IgG2a (Fig. 7b, right panel). Mice immunized with EV76 produced abundant IgG antibody to F1 antigen, but relatively little to LcrV (Fig. 7b). Mice homologously prime-boosted with rLVS Δ*capB*/Yp i.n. or heterologously prime-boosted with rLVS Δ*capB*/Yp – rLm/Yp (i.n./i.n. or i.n./i.m.) also developed T-cell mediated immunity, as evidenced by IFN-γ secretion by lung and spleen cells in response to F1 and/or LcrV (Fig. S10b, lower panels).

Mice immunized three times i.d. with rLVS Δ*capB*/Yp (Group D) survived significantly longer than sham-immunized mice (*P* = 0.03); mice primed-boosted with rLVS Δ*capB*/Yp - rLm/Yp (Group F) also survived longer than sham-immunized mice, although the difference was not statistically significant (Fig. 7c). As in the previous experiment shown in Fig. 6, the amount of serum antibody specific to F1, but not to LcrV, was highly correlated with the mean survival time three weeks post-challenge (*P* < 0.001, *P* < 0.0001 and *P* < 0.0001 for IgG, IgG1 and IgG2a, respectively) (Fig. 7d). These results indicate that systemic homologous priming-boosting with rLVS Δ*capB*/Yp induces strong protective immunity against *Y. pestis* CO92 respiratory challenge and that vaccine efficacy is correlated with F1-specific antibody.

### Potent protective immunity against pneumonic tularemia

**Figure 8:**
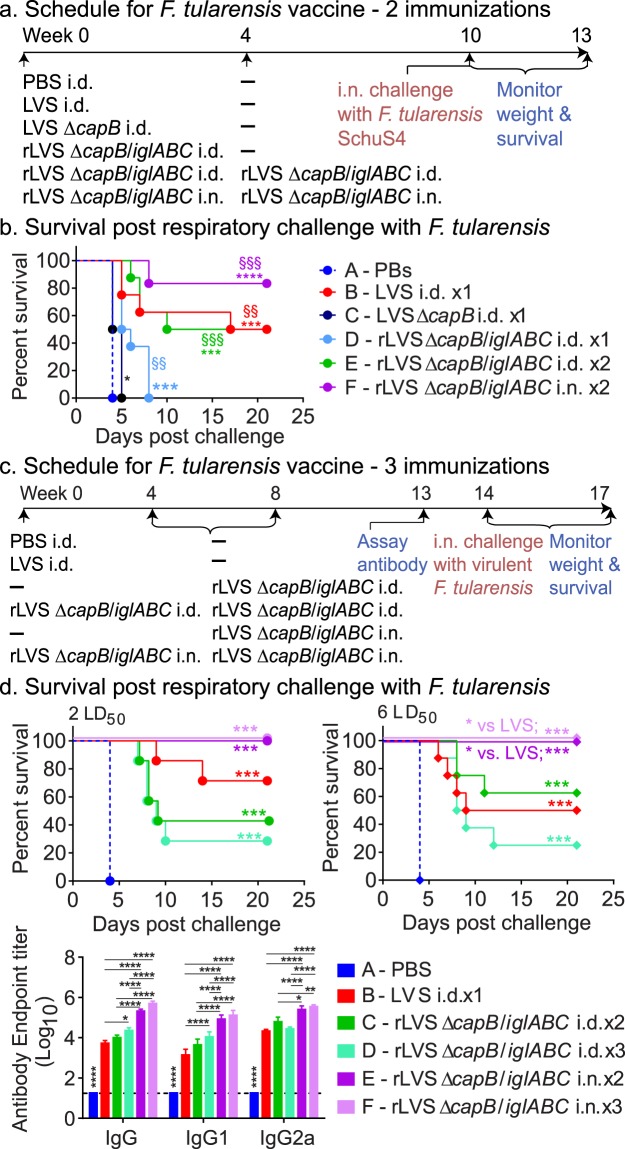
Two immunizations by homologous priming-boosting with rLVS Δ*capB/iglABC* induces strong protection against respiratory challenge with the virulent *F. tularensis* Schu S4 strain. (**a**) Schedule - Experiment VI. Mice (n = 8/group) were immunized once with PBS (Sham), 10^4^ CFU LVS, 10^6^ CFU LVS Δ*capB* vector, or 10^6^ CFU rLVS Δ*capB*/*iglABC*; or twice with 10^6^ CFU rLVS Δ*capB*/*iglABC*; challenged i.n. with *F. tularensis* Schu S4 (10 CFU, ~10 LD_50_) at Week 10; and monitored for signs of illness, weight change, and death for 3 weeks, as indicated. (**b**) Survival after vaccination and challenge – Experiment VI. The survival curve of each vaccinated group is compared with that of the Sham (Group A) or LVS Δ*capB* vector (Group C) group by the log-rank test (Mantel-cox); *P* values for vaccine groups that are significantly different from the Sham or LVS Δ*capB* vector control group are marked with asterisk(s) and multiple “§”, respectively, color-coded to the color of the vaccine symbol. *P < 0.05; ***P < 0.001; and ****P < 0.0001 vs. Group A (Sham); ^§§^*P* < 0.01; ^§§§^*P* < 0.001 vs. Group C (vector control). (**c**) Schedule - Experiment VII. Mice (n = 8/group) were immunized once with PBS (Sham), 10^4^ CFU LVS, or 10^6^ CFU rLVS Δ*capB*/*iglABC* three times at Weeks 0, 4, and 8, or twice at Weeks 4 and 8, bled at Week 13, and challenged i.n. with 2 CFU (2 LD_50_) or 6 CFU (6 LD_50_) *F. tularensis* Schu S4 at Week 14, and monitored for 3 weeks, as indicated. (**d**) Survival after vaccination and challenge and serum antibody pre-challenge – Experiment VII. Upper panels – Survival. The survival curve after challenge with 2 LD_50_ (left panel) or 6 LD_60_ (right panel) of each vaccinated group is compared with that of either the sham- or LVS-immunized group by the log-rank test (Mantel-cox); *P* values that are significantly different from the control group are marked with asterisk(s) color-coded to the color of the vaccine symbol. ***P < 0.001 vs. Group A (Sham); *P < 0.05 vs. Group B (LVS). Lower panel- Serum antibody after vaccination. Sera were assayed for IgG and subtypes IgG1 and IgG2a specific to heat-inactivated LVS. Data are mean + SEM of serum antibody endpoint titer for n = 8 per group. Differences among individual groups were compared by two-way ANOVA with Tukey’s correction. Values that are significantly different between two groups are marked with asterisk(s) over an open horizontal line crossing above the two groups. As indicated by the asterisks above the Sham group, its titers were significantly different from all other groups. *P < 0.05; **P < 0.01; and ****P < 0.0001.

In previous studies, we have shown that heterologous priming-boosting with LVS Δ*capB* or rLVS Δ*capB* overexpressing IglC as the prime vaccine and rLm expressing *F. tularensis* IglC (rLm/*iglC*) as the booster vaccine induces potent protective immunity in mice against virulent *F. tularensis* Schu S4 respiratory challenge^18^. We have also shown that immunization with rLVS Δ*capB*/*iglABC* is highly safe and induces greater protective immunity than the parental LVS Δ*capB* vector against *F. tularensis* Schu S4 respiratory challenge^19^. However, the efficacy of homologous priming-boosting with this vaccine has not been investigated. To evaluate the efficacy of homologous priming-boosting with rLVS Δ*capB*/*iglABC*, both systemically and mucosally, we immunized BALB/c mice once or twice i.d. or i.n. with this vaccine, as indicated in Fig. 8a. Control mice were sham-immunized with PBS, immunized i.d. with the unlicensed LVS vaccine (which is highly lethal by the i.n. route), or i.d. with the LVS Δ*capB* vector. Mice were challenged with 10 CFU *F. tularensis* Schu S4 (~10 LD_50_) at Week 10 and monitored for survival for 3 weeks. All immunized mice survived significantly longer than the sham-immunized mice (*P* = 0.03 for LVS Δ*capB* and *P* = 0.001 or 0.0001 for all other groups vs. sham-immunized mice). Mice immunized i.d. once (Group D) or twice (Group E) or i.n. twice (Group F) with rLVS Δ*capB*/*iglABC* survived significantly longer than mice immunized once with the LVS Δ*capB* vector (*P* = 0.006, *P* = 0.0001, and *P* = 0.0005, respectively); mice immunized i.d. or i.n. twice with rLVS Δ*capB*/*iglABC* (Groups E and F) had survival times equivalent to or greater than that of mice immunized with LVS (Group B); differences were not statistically significant (Fig. 8b).

In a subsequent experiment comparing two vs. three doses of rLVS Δ*capB*/*iglABC*, we immunized mice i.d. or i.n. twice or three times with rLVS Δ*capB*/*iglABC*; control mice were immunized i.d. once with PBS (Sham) or LVS. All the mice were bled, challenged i.n. with 2 or 6 CFU (LD_50_ ≈ 1 CFU) of *F. tularensis* Schu S4, and monitored for survival for 3 weeks (Fig. 8c). Consistent with the experiment described above, all the immunized mice survived significantly longer than the sham-immunized mice (*P* < 0.001) (Fig. 8d, upper panels). Notably, 100% of mice immunized with rLVS Δ*capB*/*iglABC* i.n. either twice or three times survived challenge with 2 or 6 LD_50_, the highest survival rate among the groups; and the survival of rLVS Δ*capB*/*iglABC* immunized mice was significantly greater (*P* < 0.05) than that of LVS-immunized mice after the 6 LD_50_ challenge (Fig. 8d upper panels). Interestingly, when the rLVS Δ*capB*/*iglABC* vaccine was administered by the i.d. route, three immunizations were not more efficacious than two immunizations; differences in survival between these mice and LVS-immunized mice were not statistically significant. All the immunized mice had *F. tularensis* antigen-specific IgG antibody, balanced between IgG1 and IgG2a, at levels significantly greater than sham-immunized mice (*P* < 0.0001) (Fig. 8d, lower panels). Mice immunized i.d. three times with rLVS Δ*capB/iglABC* (group D) had antibody (IgG and IgG1) level significantly greater than LVS-immunized mice (*P* < 0.05 and *P* < 0.0001, resp.). Mice immunized with rLVS Δ*capB/iglABC* i.n. twice (group E) or three times (group F) had antibody (IgG, IgG1, and IgG2a) levels significantly higher than mice immunized i.d. once with LVS (*P* < 0.0001) or i.d. twice or three times with rLVS Δ*capB/iglABC* (*P* < 0.05 – *P* < 0.0001) (Fig. 8d, lower panel). While not examined herein, a previous study demonstrated that rLVS Δ*capB/iglABC* also induces strong cell-mediated immune responses, which play a dominant role in host defense against *F. tularensis*^19^. These results show that systemic and especially mucosal homologous priming-boosting with rLVS Δ*capB*/*iglABC* induces strong protective immunity against virulent *F. tularensis* Schu S4 respiratory challenge.

## Discussion

In this study, we report single platform vaccines including homologous LVS Δ*capB-*vectored vaccines and heterologous LVS Δ*capB* and Lm-vectored vaccines against three Tier I pathogens, the causative agents for anthrax, plague and tularemia. We show that LVS Δ*capB*- and Lm-vectored vaccines express recombinant *B. anthracis*, *Y. pestis*, and *F. tularensis* immunoprotective proteins on solid agar or in broth, and in macrophage-like cells *in vitro;* the shuttle plasmids for antigen expression in LVS Δ*capB* vectored vaccines are stable after passage in broth, macrophages, and mice. All vaccines are safe in mice after systemic (i.d. or i.m.) or mucosal (i.n.) immunization. Homologous priming-boosting with LVS Δ*capB*-vectored *B. anthracis*, *Y. pestis*, or *F. tularensis* vaccines administered mucosally or systemically induces potent antigen-specific humoral and T cell-mediated (including both CD4+ and CD8+) immune responses, known to be important for long-lasting potent immunoprotection against the target pathogens of anthrax^34–36^, plague^20,37,38^, and tularemia^39–43^ in animal models, and potent protective immunity against respiratory challenge with lethal doses of these pathogens. Protection against anthrax was far superior to that obtained with the licensed AVA vaccine and protection against tularemia was comparable to or greater than that obtained with the toxic and unlicensed LVS vaccine. Heterologous priming-boosting with LVS Δ*capB*- and Lm-vectored *B. anthracis* and *Y. pestis* vaccines also induced potent protective immunity against respiratory challenge with virulent *B. anthracis* spores and *Y. pestis*.

Studies on the immunity induced by anthrax vaccines, mostly comprising PA and LF antigens or their immunodominant domains, have focused on serum antibody (mostly Th2 biased) and toxin neutralization antibody; the role of T cell mediated immunity has not been widely investigated^12,44^. Thus, the cumbersome immunization regimen for the licensed AVA vaccine (consisting primarily of PA with some LF protein) entails repeated boosting to induce sufficient toxin neutralizing activity for protection. With respect to a potential role for cell-mediated immunity, Glomski *et al*. reported that IFN-γ-producing CD4+ lymphocytes, but not humoral immune responses, mediate spore-induced immunity to capsulated *B. anthracis*^36^. Also, in this regard, Altman has reported that patients recovered from cutaneous anthrax, who anecdotally exhibit long-term protection from subsequent infection, show high frequencies of CD4+ T cells in response to PA and LF^34^. In our study, the AVA vaccine, which gave very poor protection against respiratory challenge with *B. anthracis*, induced strong antibody responses and toxin neutralization activity but essentially no T-cell responses to PA, and neither humoral nor T-cell responses to LF. In contrast, homologous prime-boost vaccination with rLVS Δ*capB*/Ba and heterologous prime-boost vaccination with rLVS Δ*capB*/Ba – rLm/Ba expressing an LFnPAc fusion protein, which gave strong protection against *B. anthracis* respiratory challenge, not only induced PA- and LF-specific serum IgG, dominated by Th1-type IgG2a, and toxin neutralizing antibody, but also induced strong T-cell responses, including IFNγ-, TNFα-, and/or IL2-expressing multifunctional CD4+ T cells, suggesting a role for T-cell mediated immunity in addition to humoral immunity in protection against anthrax. In our study, the antibody titer to LF, but not to PA, correlated with protection. The lack of correlation between protection and anti-PA antibody in our study is consistent with results of some studies^45,46^ but contrasts with the results of others^47–49^.

Currently there is no licensed vaccine against *Y. pestis*. Vaccines studied preclinically include live attenuated *Y. pestis* vaccines, such as EV76; subunit vaccines based primarily on F1 and LcrV antigens; and live attenuated heterologous bacterial (*Salmonella* and *Yersinia pseudotuberculosis*) or viral (adenovirus, modified vaccinia Ankara, or bacteriophage) vectors expressing F1 and/or LcrV^50–55^. These vaccines have their advantages and disadvantages. Generally speaking, live attenuated *Y. pestis* vaccines, while effective, induce serious local and systemic reactions^55^; subunit vaccines are effective but do not induce high levels of cell-mediated immunity, likely important for long-lasting protection^56^; heterologous bacterial vectors are safe but have not been as effective as subunit vaccines^57^; and viral vectored vaccines are effective but may be limited by preexisting immunity. Immunology studies have shown that antibodies to F1 and LcrV provide short-term protective immunity against challenge with *Y. pestis;* however, F1-LcrV-specific T cell responses with a preferential Th1 polarization are also critical for protection against *Y. pestis*^55,57^. Our study shows that homologous prime-boost vaccination (two or three doses) with rLVS Δ*capB*/Yp or heterologous prime-boost vaccination with rLVS Δ*capB*/Yp-rLm/Yp induces significantly elevated serum antibody to F1-LcrV, balanced between IgG1 and IgG2a, elevated IFN-γ secretion by spleen and lung cells, and partial protection against pneumonic challenge with virulent *Y. pestis*; protection correlates with serum antibody to F1. Protection was not as great as that induced by immunization with the toxic and unlicensed EV76 vaccine, which induced much higher levels of antibody to F1 antigen; the suboptimal protective immunity induced by rLVS Δ*capB*/Yp and/or rLm/Yp vaccines may have been due to the relatively poor immunogenicity of their F1-LcrV fusion protein construct. It has been shown that the mutated form of F1, F1mut, folds into a monomer, rather than polymerizing as a linear fiber, enhancing its immunogenicity^11,54^. Potentially, the efficacy of our vaccines can be substantially enhanced by similarly expressing F1mut as well as by expanding the antigen repertoire by the addition of other immunoprotective antigens.

As with plague, there are no licensed vaccines against tularemia. The most promising vaccine candidates being studied preclinically, like the unlicensed LVS vaccine, are live attenuated *Francisella* vaccines, including our LVS Δ*capB* vectored vaccine, derived ultimately from *F. tularensis* subsp. *holarctica*^17^; vaccines derived from the nonhuman pathogen *F. novicida*^58^; and vaccines derived from the highly virulent human pathogen *F. tularensis* subsp. *tularensis* Schu S4 strain^59–61^. Single deletional mutants of *F. tularensis* Schu S4 are effective, comparable to LVS and the rLVS Δ*capB* vaccines, but safety considerations dictate the need for at least one additional major attenuating deletion; thus far, such additional deletions have resulted in impotent vaccines^62^. In contrast, our rLVS Δ*capB/iglABC * vaccine is highly potent; has three major attenuating deletions; is >10,000-fold less virulent than LVS in the mouse model; is unmarked, i.e. devoid of antibiotic resistance genes; and has an excellent safety profile^17^.

Our previous studies have shown that systemic (i.d.) vaccination with a single dose of rLVS Δ*capB*/*iglABC* induces potent CD4+ and CD8+ T cell immune responses, Th1-dominated serum antibody, and partial protection against respiratory challenge with *F. tularensis* Schu S4^19^. Our study here shows that systemic i.d. vaccination with just two doses rLVS Δ*capB*/*iglABC* induces substantial protection to pneumonic challenge with *F. tularensis* Schu S4 strain, equivalent to the LVS vaccine; mucosal (i.n.) vaccination with either two or three doses of rLVS Δ*capB*/*iglABC* provides 100% protection.

To our knowledge, our platform is the first to demonstrate efficacy against three Tier 1 Select Agents. Vaccines have previously been described against two pathogens, including a vaccinia-based vaccine against smallpox and anthrax^32^ and subunit vaccines against anthrax and plague^11,63^. Among the strategies tested in our proof of principle studies – (a) LVS Δ*capB*- vs. Lm-vectored vaccines; (b) homologous vs. heterologous prime-boost vaccination; (c) mucosal vs. systemic (i.d. for rLVS Δ*capB* and i.m. for rLm) vaccine administration; (d) one vs. two vs. three immunization doses – we found that three homologous i.d. vaccinations with the LVS Δ*capB*-vectored *B. anthracis*, *Y. pestis*, and *F. tularensis* vaccines is a strategy that works well for all three target pathogens. This strategy has the major advantage of being a single vector platform, induces strong antigen-specific T-cell and humoral immune responses, and provides potent efficacy against all three pathogens. In the case of *F. tularensis*, mucosal i.n. delivery of the rLVS Δ*capB*/*iglABC* vaccine was somewhat superior to i.d. delivery of this vaccine; however, the i.n. route raises safety issues and administering this one vaccine by that route and the anthrax and plague vaccines by the i.d. route would preclude concurrent immunization of all three vaccines together. In the case of *B. anthracis*, the heterologous prime-boost vaccination strategy was also highly effective; however, this approach would require development of two vaccines based upon different vectors and likely require administration by two different routes, a significant disadvantage in terms of cost and complexity of development, manufacture, and regulatory approval, and ease of clinical administration.

With respect to safety in humans, both the LVS and the Lm Δ*actA* Δ*inlB* parental vectors have established safety profiles in humans. LVS, which retains significant virulence in animals and shows residual toxicity in humans, is the only tularemia vaccine demonstrated efficacious in humans. In comparison with its wild-type *F. tularensis subsp. holarctica* parent, LVS has two major attenuating deletions, FTT0918 (virulence determinate of SCHU S4) and *pilA*^64,65^, and as noted above, our LVS Δ*capB* vector has a third major attenuating deletion, *capB*, rendering it >10,000 fold less virulent for mice than LVS by the i.n. route. The Lm Δ*actA* Δ*inlB* vector has been shown to be safe in humans^23^, and the additionally modified Lm Δ*actA* Δ*inlB prfA*(G155S) vector retains the attenuation of the parental Lm Δ*actA* Δ*inlB* vector in mice^25^ while providing significantly enhanced antigen-specific T-cell mediated immune responses^24,25^. Our study shows that the LVS Δ*capB*- and Lm Δ*actA* Δ*inlB prfA*(G155S)-vectored *F. tularensis*, *B. anthacis*, and *Y. pestis* vaccines are safe and efficacious in BALB/c mice. Further validation of genetic stability and safety of the vaccines will be required prior to human studies.

In summary, our live attenuated single vector vaccine platform elicits robust humoral and T-cell mediated immune responses and protective immunity against three target pathogens – the agents of anthrax, plague, and tularemia – and overcomes the problems of poor immunogenicity and lack of immunological memory often associated with subunit vaccines against these pathogens. As already noted, a single vector platform vaccine has numerous advantages in terms of production cost, regulatory approval, ease of administration, and patient acceptability. This vaccine platform can be extended readily to cover other pathogens including other Tier 1 Select Agents such as *Burkholderia pseudomallei* and *Burkholderia mallei*.

## Materials and Methods

### Cell line, bacteria, and vaccines

Mouse macrophage-like J774A.1 cells (ATCC TIB-67) and human macrophage-like THP-1 cells (ATCC TIB-202) were negative for mycoplasma contamination and cultured as described previously^15,24^*. F. tularensis* Live Vaccine Strain (LVS) was obtained from the Centers for Disease Control and Prevention (Atlanta, Ga.). *B. anthracis* AVA (Anthrax vaccine adsorbed) vaccine was obtained from BEI Resources. *Y. pestis* attenuated EV76 strain was obtained from Centers for Disease Control and Prevention, Fort Collins, CO. Virulent strains of *B. anthracis* (Ames spores), *Y. pestis* (CO92), and *F. tularensis* (Schu S4) were originally obtained from BEI Resources, stored at −80 °C, and used in animal challenge experiments at Colorado State University (CSU) only. The LVS, LVS Δ*capB* vector, recombinant LVS Δ*capB* vaccine, Lm vector, and recombinant Lm (rLm) vaccine stocks were prepared, stored and used as described previously by us^15,17,18^.

### Mice

Six to eight week old female BALB/c mice were purchased from Charles River Laboratory (Wilmington, MA) or Taconic (Hudson, NY) and randomly assigned to experimental groups. Animals for immunology studies and for efficacy studies prior to challenge were maintained in a specific-pathogen-free animal facility and used according to protocols approved by the UCLA and CSU Institutional Animal Care and Use Committees. After challenge with virulent *B. anthracis, Y. pestis*, or *F. tularensis*, animals were maintained in a BSL3 facility at CSU and used according to protocols approved by the CSU IACUC.

### Proteins, antibodies, and vaccine controls

We obtained the following reagents through the NIH NIAID Biodefense and Emerging Infections Research Resources Repository: genomic DNA from *B. anthracis*, strain Sterne BA695 (Δlef243), NR-9540; genomic DNA from *Y. pestis* strain CO92, DD-494; monoclonal antibody to PA, AB-BA-PA-MAB1; goat polyclonal antibody to LcrV, NR-31022; goat polyclonal antibody to F1-Antigen, NR-31024; PA protein, NR-3780; Lethal Factor (LF-HMA) protein, NR-4368; LcrV protein, NR-32875; F1 protein, NR-44223; F1-LcrV monomer protein, NR-2562; Anthrax Vaccine Adsorbed (AVA) (BioThrax™), NR-2642; and AVA pooled human plasma, NR-28672. Rabbit polyclonal antibody to *F. tularensis* Bacterioferritin (Bfr) was produced in our laboratory^31^.

### Construction and verification of rLVS Δ*capB* vaccines expressing *B. anthracis, Y. pestis*, or *F. tularensis* fusion proteins encoded by genes amplified from *B. anthracis*, *Y. pestis*, and *F. tularensis* genomic DNAs, respectively

Using LVS Δ*capB* and pFNL-derived shuttle vectors^66^, we constructed recombinant LVS Δ*capB* (rLVS Δ*capB*) vaccine candidates, rLVS Δ*capB*/*bfr*-*iglABC* (rLVS Δ*capB*/*iglABC*)^19^, rLVS Δ*capB*/Ba, and rLVS Δ*capB*/Yp, using a strategy similar to one published previously^19^. Specifically, we constructed the shuttle plasmid for expressing *B. anthracis* fusion protein LFnPAc, pFNL/*bfr*-BaLFnPAc(GGSG), by replacing the *gro-gfp* expression cassette in pFNLTB6 *gro-gfp* (*Kan*^*R*^)^66^ with the *bfr*-BaLFnPAc(GGSG) expression cassette comprising the *F. tularensis bacterioferritin* promoter (upstream of FTT_1441, *bfr*, amplified from the genomic DNA of an clinical isolate of SCHU S4 strain), a Shine-Dalgarno sequence, a 6-nucleotide spacer, followed by the coding sequence for *B. anthracis* LFn^26,27^ and PAc^12^ separated by a GGSG linker (LFnPAc). The coding sequence for LFnPAc was amplified by two-step overlap PCRs. First, we amplified the coding sequences for LFn and PAc by using the genomic DNA of *B. anthracis* (BEI NR-9540) and primer pairs LF_Fw1 (TAACAATAGGAGGTACGTAATGGCGGGCGGTCATGGTGATG) and LFn_Rv (TGTTTGTTGATCGAAATTACCAGAACCACCTAGATTTATTTCTTGTT) for LFn, and primer pairs PAc_Fw (AACAAGAAATAAATCTAGGTGGTTCTGGTAATTTCGATCAACAAACA) and PA_Rv1 (TGAAACGAG CTAGTGGATCCTTATCCTATCTCATAGCCTTTTT) for PAc, respectively. Secondly, we used the PCR products of LFn and PAc and primer pairs LF_Fw1 and PA_Rv1 to amplify the coding sequence for fusion protein LFnPAc, which was subsequently cloned into the pFNL-derived shuttle vector. We verified the inserted sequence by restriction analysis and nucleotide sequencing, electroporated the resultant shuttle plasmid into LVS Δ*capB*, and selected clones that were kanamycin-resistant and stably expressed the targeted antigens shown by Western blotting. Similarly, we constructed rLVS Δ*capB*/Yp carrying the shuttle plasmid pFNL/*omp*-YpF1V(GGSG) for expressing the *Y. pestis* fusion protein of F1 (170 aa) and LcrV (326 aa) separated by the GGSG linker (F1V) driven by the *F. novicida* outer membrane promoter (upstream of FTN_1451, *omp*, amplified from *F. novicida* genomic DNA). The coding sequence for F1 and LcrV were amplified from the genomic DNAs of *Y. pestis* (BEI DD-494) by using primer pair F1_Fw1 (GACTAAAAGGAGGTACGTAATGAAAAAAATCAGTTCCGTTAT) and F1_Rv (TGTTCGTAGGCTCTAATCAT*ACCAGAACCACC*TTGGTTAGATACGGTTAC) for F1 and primer pair LcrV_Fw (GTAACCGTATCTAACCAAGGTGGTTCTGGTATGATTAGAGCCTACGAACA) and LcrV_Rv1 (CGAGCTAGTGGATCCTCATTTACCAGACGTGTCATCTA) for LcrV, respectively.

### Construction and verification of attenuated recombinant *Listeria monocytogenes* vaccines expressing *B. anthracis, Y. pestis*, and *F. tularensis* fusion proteins

Using Lm Δ*actA* Δ*inlB* Δ*uvrAB prfA*(*G155S*) (Lm Δ*actA* Δ*inlB prfA*, generously provided by J. Skoble of Aduro Biotech, previously Anza Therapeutics)^25,33,67^ as a vector, we constructed recombinant Listeria-vectored vaccine candidates expressing immunogenic fusion proteins of *B. anthracis*, *Y. pestis*, or *F. tularensis*, using methodology described previously by us and others^15,22,24,68^. Briefly, we amplified the encoding sequence for the fusion protein of *B. anthracis* LFnPAc and *Y. pestis* F1V from the above described *F. tularensis* shuttle plasmids by PCR with primer pairs LF_Fw2 (AGGTGGATCCATGGCGGGCGGTCATGGTG) and PA_Rv2 (CGGTGGCGGCCGCTTATCCTATCTCATAGCCTTTTTTA) for LFnPAc and F1_Fw2 (CGAGGGATCCATGAAAAAAATCAGTTCCGTTAT) and LcrV_Rv2 (ATATGCGGCCGCTCATTTACCAGACGTGTCATCTA) for F1V, respectively, ligated them with either the Lm *hly* promoter and the coding sequence for the listeriolysin O (encoded by *hly*) signal sequence (LLOss) or the Lm *actA* promoter and the coding sequence for the ActA N-terminal 100 amino acids (ActAN), and cloned into a phage-based *Listeria* site-specific integration vector derived from pPL2 (kindly provided by J. Skoble)^68^. We subsequently integrated the resultant plasmid into the 3′ end of tRNA^arg^ on the bacterial chromosome of the recipient Lm Δ*actA* Δ*inlB prfA* strain from the donor SM10 strain carrying the integration plasmid through conjugation to obtain rLm vaccines. We confirmed all the molecular plasmid constructs by nucleotide sequencing and verified the final recombinant *L. monocytogenes* strains by colony PCR for chromosomal integration and by Western blotting for heterologous protein expression.

### Heterologous protein expression by and growth kinetics of LVS Δ*capB*- and Listeria-vectored vaccines in broth culture and in infected macrophage-like cells

To assess protein expression by rLVS Δ*capB* vaccines grown on agar, we grew each of the vaccine stocks on Chocolate agar, selected single colonies, lysed them in SDS buffer, applied the lysates to SDS-PAGE, and analyzed protein expression by Western blotting. Secreted proteins in the supernate of Brain Heart Infusion (BHI) broth culture of rLm vaccines were precipitated by the TCA-acetone method and analyzed by Western blotting. Monoclonal antibodies specific to *B. anthracis* PA (BEI, DD-9) and goat polyclonal antisera specific to *Y. pestis* LcrV (BEI, NR-31022) were used as primary antibody in Western blotting. To assay protein expression of LVS Δ*capB*-vectored vaccines in macrophage-like cells, we seeded monocytic THP-1 cells at 3 × 10^5^ cells/well on 24-well plates and differentiated them in the presence of PMA for 3 days. Vaccine vector (LVS Δ*capB*) and rLVS Δ*capB* vaccines were grown on Chocolate agar supplemented without (vector) or with (vaccines) kanamycin (7.5 μg/ml) for 3 days. The differentiated THP-1 cells were left uninfected or infected with rLVS Δ*capB*/Ba or rLVS Δ*capB*/Yp opsonized with human serum at a multiplicity of infection (MOI) of 10:1 (bacteria: cell) and incubated at 37 °C for 1 h. The cells were then washed with RPMI three times and incubated with complete RPMI supplemented with gentamycin (0.1 μg/ml) to inhibit extracellular bacterial growth. At 24 h post infection, medium was removed from wells; cells were lysed, and cell lysates analyzed by Western blotting using a mixture of monoclonal antibody to *B. anthracis* PA antigen and goat polyclonal antibody to *Y. pestis* LcrV antigen. Protein expression of Lm-vectored vaccines in macrophage-like cells was assayed as described previously^15,24^. Growth kinetics of LVS Δ*capB-* and Lm-vectored vaccines in broth and in infected macrophages were assayed as described in the Supplemental Information (legends to Figs S2 and S3, resp.) and as published by us previously^15,19,24^.

### Immune response analysis

Groups of 4–8 BALB/c mice were immunized as indicated in Figs 2a, 3a, 4a, 6a and 8a,c, Figs S7a and S10a. In experiments studying immunology and efficacy of *B. anthracis, Y. pestis* and *F. tularensis* vaccines, mice were bled one week prior to challenge. In experiments studying immunology only, mice were bled at one week post rLm boosting and subsequently euthanized; spleens and lungs removed; single cell suspensions of spleen and lung cells prepared and suspended in T cell medium; and cells enumerated as described previously^19^. Serum was isolated and stored at −80 °C until use. Serum antibody, *in vitro* stimulation and production of IFN-γ and IL-4 by murine immune lung and spleen cells, and intracellular cytokine staining of lung and spleen cells for flow cytometry analysis were assayed as published by us previously^15,19,24^.

#### Serum antibody

Sera were tested for IgG antibody response by enzyme-linked immunosorbent assay (ELISA) using standard procedures^15^. Briefly, ninety-six-well microtiter plates were coated with recombinant protein PA (BEI, NR-3780), LF (NR-4268), F1 (NR-44223), LcrV (NR-32875), or F1-LcrV monomer (NR-2563) at 1 µg/ml each, or heat-inactivated LVS (HI-LVS, 2 × 10^7^/ml) diluted in carbonate buffer overnight at 4 °C and afterwards processed at ambient temperature. The plates were washed three times with 0.05%Tween20-PBS, blocked in 3% BSA-PBS for 3 h, incubated with each serum sample serially diluted 2-fold twelve times at a starting dilution of 1:20 or 1:50 in 1% BSA-PBS for 90 min, and washed again. Bound antibody was detected by using alkaline phosphatase-conjugated goat anti-mouse IgG (Sigma, St. Louis, MO), subtypes IgG1 (Sigma), IgG2a (Abcam), IgG2b (sigma), or IgG3 (Sigma) diluted in 1% BSA-PBS and incubating for 90 min. Plates were developed with 100 µl of p-nitrophenylphosphate substrate (BioRad), and the *A415* was read using a multiscan microplate reader (iMark, BioRad). The results are presented as the mean antibody endpoint titer and SE of the mean (SEM). Antibody endpoint titer is defined as the mean log dilution that yields an OD greater than the mean OD of Sham sera plus three standard deviations at the same serum dilution.

#### *In vitro* stimulation and production of IFN-γ and IL-4 by murine immune splenocytes

A single cell suspension of 1.0 × 10^5^ splenocytes or lung cells per well was seeded in U-bottom 96-well plates and incubated with T-cell medium alone, or T-cell medium supplemented with 2 µg/mL of recombinant PA, LF, F1, LcrV, or F1-LcrV monomer for three days. After a 3-day incubation, the culture supernatant fluid was collected, cell debris removed by centrifugation, and the supernatant fluid stored in assay diluent (BD Biosciences) at −80 °C until use. The production of mouse IFN-γ and IL-4 in the culture supernatant fluid was assayed using a mouse cytokine EIA kit (BD Biosciences)^24^.

#### *In vitro* stimulation and intracellular cytokine staining for flow cytometry analysis

A single cell suspension of 7.5 × 10^5^ lung cells or 1.5 × 10^6^ splenocytes per well was seeded in U-bottom 96-well plates and stimulated with 2 µg/mL of recombinant PA or LF, or 2 × 10^7^/ml of HI-LVS in the presence of anti-CD28 monoclonal antibody (Clone 37.51) for a total of 6 h, and processed for Flow Cytometry analysis as described previously by us^19,24^. The frequencies of live CD4+ and CD8+ T cells producing any of the 15 possible combinations of four cytokines (IFN-γ, TNF-α, IL-2, and IL-17A) were uniquely distinguished using logic combinations of the gates for each cytokine and FACSDiva (BD) software. Background frequencies of cells producing cytokines without antigen stimulation were subtracted.

### Protective efficacy

Efficacy of rLVS Δ*capB*- and rLm-vectored *B. anthracis*, *Y. pestis*, and *F. tularensis* vaccines was studied at CSU similarly to what was previously described by us^17–19^. Virulent strains of *B. anthracis* (Ames), *Y. pestis* (CO92), and *F. tularensis* (Schu S4) were originally obtained from BEI Resources, stored at −80 °C, and used to make working stocks for animal challenge experiments at CSU. Briefly, *Y. pestis* was grown to log phase at 37 °C in BHI broth and *F. tularensis* was grown on modified Mueller-Hinton agar plates and colonies collected into Mueller-Hinton broth. For both *Y. pestis* and *F. tularensis*, glycerol was added to the harvested bacterial suspensions to 15% (v/v) and the suspensions frozen in aliquots at −80 °C. *B. anthracis* spores were prepared using published procedures^69^, resuspended in PBS, and frozen in in aliquots at −80 °C. We pre-determined that the 50% lethal dose (LD_50_) of *B. anthracis* (Ames) spores, *Y. pestis* CO92, and *F. tularensis* Schu S4 administered intranasally (i.n.) in BALB/c mice is approximately 40,000, 250, and 1 CFU, respectively. Mice were sham-immunized or immunized with 2 or 3 doses of rLVS Δ*capB* or rLm vaccines (homologous prime-boost vaccination) or primed-boosted with rLVS Δ*capB* - rLm vaccines (heterologous prime-boost vaccination), challenged i.n. with virulent *B. anthracis* (Ames) spores, *Y. pestis* CO92, or *F. tularensis* Schu S4, weighed, and monitored for illness and death for 3 weeks, as indicated. Mice that met predetermined humane endpoints for euthanasia were euthanized and counted as a death. Mean survival time was calculated by dividing the sum of the surviving days of all animals by the total number of animals examined, with animals surviving until the end of the experiment given a time of 21 days, when the experiment was terminated.

### Statistical analyses

The sample sizes for assaying vaccine clearance and dissemination (4/group/time point), immune responses after vaccination (4/group), and efficacy (8/group) after challenge were estimated based on previous and pilot studies (GraphPad StatMate 2.0). Means and SE of the mean (SEM) of serum antibody endpoint titer, cytokine production, and frequencies of cytokine-producing CD4+ and CD8+ T cells were reported, and means compared across groups by ANOVA with Tukey’s correction for multiple comparisons test using GraphPad Prism, 6.04 (San Diego, CA). A log-rank analysis (Mantel-Cox test) (Prism 6.04) was used to determine the significance of differences in survival curves among mice in vaccinated and control groups. Linear regression was used to obtain values for the slope and intercept and the correlation coefficient (R^2^) between pre-challenge serum antibody endpoint titer and post-challenge mean survival time (days) at 21 days post-challenge.

### Data availability

All data supporting the findings of this study are available within the article and its supplementary information files or from the corresponding author upon request.

## Electronic supplementary material

Supplementary Information

## References

[1] Jernigan DB (2002). Investigation of bioterrorism-related anthrax, United States, 2001: epidemiologic findings. Emerg Infect Dis.

[2] Rosenzweig JA (2011). Progress on plague vaccine development. Appl Microbiol Biotechnol.

[3] Matyas BT, Nieder HS, Telford SR (2007). Pneumonic tularemia on Martha’s Vineyard: clinical, epidemiologic, and ecological characteristics. Ann N Y Acad Sci.

[4] Alibek K. *Biohazard: the chilling true story of the largest covert biological weapons program in the world, told from the inside by the man who ran it*. Random House, Inc. (1999).

[5] Inglesby TV (2000). Plague as a biological weapon: medical and public health management. Working Group on Civilian Biodefense. JAMA.

[6] Peterson JW (2010). Protection Afforded by Fluoroquinolones in Animal Models of Respiratory Infections with Bacillus anthracis, Yersinia pestis, and Francisella tularensis. Open Microbiol J.

[7] Pavlov VM, Mokrievich AN, Volkovoy K (1996). Cryptic plasmid pFNL10 from Francisella novicida-like F6168: the base of plasmid vectors for Francisella tularensis. FEMS Immunol Med Microbiol.

[8] Athamna A (2004). Selection of Bacillus anthracis isolates resistant to antibiotics. J Antimicrob Chemother.

[9] Galimand M (1997). Multidrug resistance in Yersinia pestis mediated by a transferable plasmid. N Engl J Med.

[10] Titball RW, Oyston PC (2003). A vaccine for tularaemia. Expert opinion on biological therapy.

[11] Tao P (2017). A Bivalent Anthrax-Plague Vaccine That Can Protect against Two Tier-1 BioterrorPathogens, Bacillus anthracis and Yersinia pestis. Frontiers in immunology.

[12] Baillie LW (2010). An anthrax subunit vaccine candidate based on protective regions of Bacillus anthracis protective antigen and lethal factor. Vaccine.

[13] Friedlander AM, Little SF (2009). Advances in the development of next-generation anthrax vaccines. Vaccine.

[14] Galen JE (2015). A bivalent typhoid live vector vaccine expressing both chromosome- and plasmid-encoded Yersinia pestis antigens fully protects against murine lethal pulmonary plague infection. Infect Immun.

[15] Jia Q, Lee BY, Clemens DL, Bowen RA, Horwitz MA (2009). Recombinant attenuated Listeria monocytogenes vaccine expressing Francisella tularensis IglC induces protection in mice against aerosolized Type A F. tularensis. Vaccine.

[16] Huntley JF (2008). Native outer membrane proteins protect mice against pulmonary challenge with virulent type A Francisella tularensis. Infect Immun.

[17] Jia Q (2010). A Francisella tularensis live vaccine strain (LVS) mutant with a deletion in capB, encoding a putative capsular biosynthesis protein, is significantly more attenuated than LVS yet induces potent protective immunity in mice against F. tularensis challenge. Infect Immun.

[18] Jia Q, Bowen R, Sahakian J, Dillon BJ, Horwitz MA (2013). A heterologous prime-boost vaccination strategy comprising the Francisella tularensis live vaccine strain capB mutant and recombinant attenuated Listeria monocytogenes expressing F. tularensis IglC induces potent protective immunity in mice against virulent F. tularensis aerosol challenge. Infect Immun.

[19] Jia Q (2016). Francisella tularensis Live Vaccine Strain deficient in capB and overexpressing the fusion protein of IglA, IglB, and IglC from the bfr promoter induces improved protection against F. tularensis respiratory challenge. Vaccine.

[20] Lin JS (2010). TNFalpha and IFNgamma contribute to F1/LcrV-targeted immune defense in mouse models of fully virulent pneumonic plague. Vaccine.

[21] Szaba FM (2014). TNFalpha and IFNgamma but not perforin are critical for CD8 T cell-mediated protection against pulmonary Yersinia pestis infection. PLoS Pathog.

[22] Bruhn KW, Craft N, Miller JF (2007). Listeria as a vaccine vector. Microbes Infect.

[23] Le DT (2012). A live-attenuated Listeria vaccine (ANZ-100) and a live-attenuated Listeria vaccine expressing mesothelin (CRS-207) for advanced cancers: phase I studies of safety and immune induction. Clin Cancer Res.

[24] Jia Q, Dillon BJ, Maslesa-Galic S, Horwitz MA (2017). Listeria-vectored vaccine expressing the Mycobacterium tuberculosis 30 kDa major secretory protein via the constitutively active prfA* regulon boosts BCG efficacy against tuberculosis. Infect Immun.

[25] Lauer P (2008). Constitutive Activation of the PrfA regulon enhances the potency of vaccines based on live-attenuated and killed but metabolically active Listeria monocytogenes strains. Infect Immun.

[26] Pannifer AD (2001). Crystal structure of the anthrax lethal factor. Nature.

[27] Ballard JD, Collier RJ, Starnbach MN (1996). Anthrax toxin-mediated delivery of a cytotoxic T-cell epitope *in vivo*. Proc Natl Acad Sci USA.

[28] Zaide G (2011). Identification and characterization of novel and potent transcription promoters of Francisella tularensis. Appl Environ Microbiol.

[29] Gallagher LA (2007). A comprehensive transposon mutant library of Francisella novicida, a bioweapon surrogate. Proc Natl Acad Sci USA.

[30] Ludu JS (2008). Genetic elements for selection, deletion mutagenesis and complementation in Francisella spp. FEMS Microbiol Lett.

[31] Lee BY, Horwitz MA, Clemens DL (2006). Identification, recombinant expression, immunolocalization in macrophages, and T-cell responsiveness of the major extracellular proteins of Francisella tularensis. Infect Immun.

[32] Merkel TJ (2010). Development of a highly efficacious vaccinia-based dual vaccine against smallpox and anthrax, two important bioterror entities. Proc Natl Acad Sci USA.

[33] Brockstedt DG (2004). Listeria-based cancer vaccines that segregate immunogenicity from toxicity. Proc Natl Acad Sci USA.

[34] Altmann DM (2015). Host immunity to Bacillus anthracis lethal factor and other immunogens: implications for vaccine design. Expert Rev Vaccines.

[35] Chitlaru T, Altboum Z, Reuveny S, Shafferman A (2011). Progress and novel strategies in vaccine development and treatment of anthrax. Immunol Rev.

[36] Glomski IJ, Corre JP, Mock M, Goossens PL (2007). Cutting Edge: IFN-gamma-producing CD4 T lymphocytes mediate spore-induced immunity to capsulated Bacillus anthracis. J Immunol.

[37] Quenee LE, Ciletti NA, Elli D, Hermanas TM, Schneewind O (2011). Prevention of pneumonic plague in mice, rats, guinea pigs and non-human primates with clinical grade rV10, rV10-2 or F1-V vaccines. Vaccine.

[38] Lin JS, Kummer LW, Szaba FM, Smiley ST (2011). IL-17 contributes to cell-mediated defense against pulmonary Yersinia pestis infection. J Immunol.

[39] Chen W, KuoLee R, Shen H, Conlan JW (2004). Susceptibility of immunodeficient mice to aerosol and systemic infection with virulent strains of Francisella tularensis. Microb Pathog.

[40] Conlan WJ, Shen H, Kuolee R, Zhao X, Chen W (2005). Aerosol-, but not intradermal-immunization with the live vaccine strain of Francisella tularensis protects mice against subsequent aerosol challenge with a highly virulent type A strain of the pathogen by an alphabeta T cell- and interferon gamma- dependent mechanism. Vaccine.

[41] Wu TH (2005). Intranasal vaccination induces protective immunity against intranasal infection with virulent Francisella tularensis biovar A. Infect Immun.

[42] Griffin AJ, Crane DD, Wehrly TD, Bosio CM (2015). Successful protection against tularemia in C57BL/6 mice is correlated with expansion of Francisella tularensis-specific effector T cells. Clin Vaccine Immunol.

[43] Eneslatt K (2012). Signatures of T cells as correlates of immunity to Francisella tularensis. PLoS One.

[44] Boyaka PN (2003). Effective mucosal immunity to anthrax: neutralizing antibodies and Th cell responses following nasal immunization with protective antigen. J Immunol.

[45] Ivins BE, Welkos SL, Little SF, Crumrine MH, Nelson GO (1992). Immunization against anthrax with Bacillus anthracis protective antigen combined with adjuvants. Infect Immun.

[46] Turnbull PC, Broster MG, Carman JA, Manchee RJ, Melling J (1986). Development of antibodies to protective antigen and lethal factor components of anthrax toxin in humans and guinea pigs and their relevance to protective immunity. Infect Immun.

[47] Reuveny S (2001). Search for correlates of protective immunity conferred by anthrax vaccine. Infect Immun.

[48] Chen L (2014). Comprehensive analysis and selection of anthrax vaccine adsorbed immune correlates of protection in rhesus macaques. Clin Vaccine Immunol.

[49] Fay MP (2012). Anthrax vaccine-induced antibodies provide cross-species prediction of survival to aerosol challenge. Sci Transl Med.

[50] Leary SE, Griffin KF, Garmory HS, Williamson ED, Titball RW (1997). Expression of an F1/V fusion protein in attenuated Salmonella typhimurium and protection of mice against plague. Microb Pathog.

[51] Sun W, Sanapala S, Rahav H, Curtiss R (2015). Oral administration of a recombinant attenuated Yersinia pseudotuberculosis strain elicits protective immunity against plague. Vaccine.

[52] Boyer JL (2010). Protective immunity against a lethal respiratory Yersinia pestis challenge induced by V antigen or the F1 capsular antigen incorporated into adenovirus capsid. Hum Gene Ther.

[53] Brewoo JN, Powell TD, Stinchcomb DT, Osorio JE (2010). Efficacy and safety of a modified vaccinia Ankara (MVA) vectored plague vaccine in mice. Vaccine.

[54] Tao P (2013). Mutated and bacteriophage T4 nanoparticle arrayed F1-V immunogens from Yersinia pestis as next generation plague vaccines. PLoS Pathog.

[55] Feodorova VA, Motin VL (2012). Plague vaccines: current developments and future perspectives. Emerging microbes & infections.

[56] Kummer LW (2008). Antibodies and cytokines independently protect against pneumonic plague. Vaccine.

[57] Williamson ED, Oyston PC (2013). Protecting against plague: towards a next-generation vaccine. Clin Exp Immunol.

[58] Chu P (2014). Live attenuated Francisella novicida vaccine protects against Francisella tularensis pulmonary challenge in rats and non-human primates. PLoS Pathog.

[59] Reed DS (2014). Live attenuated mutants of Francisella tularensis protect rabbits against aerosol challenge with a virulent type A strain. Infect Immun.

[60] Rockx-Brouwer D (2012). Low dose vaccination with attenuated Francisella tularensis strain SchuS4 mutants protects against tularemia independent of the route of vaccination. PLoS One.

[61] Conlan JW (2011). Tularemia vaccines: recent developments and remaining hurdles. Future Microbiol.

[62] Conlan JW (2010). Differential ability of novel attenuated targeted deletion mutants of Francisella tularensis subspecies tularensis strain SCHU S4 to protect mice against aerosol challenge with virulent bacteria: effects of host background and route of immunization. Vaccine.

[63] DuBois AB, Freytag LC, Clements JD (2007). Evaluation of combinatorial vaccines against anthrax and plague in a murine model. Vaccine.

[64] Salomonsson E (2009). Reintroduction of two deleted virulence loci restores full virulence to the live vaccine strain of Francisella tularensis. Infect Immun.

[65] Twine S (2005). A mutant of Francisella tularensis strain SCHU S4 lacking the ability to express a 58-kilodalton protein is attenuated for virulence and is an effective live vaccine. Infect Immun.

[66] Maier TM (2004). Construction and characterization of a highly efficient Francisella shuttle plasmid. Appl Environ Microbiol.

[67] Yan L (2008). Selected prfA* mutations in recombinant attenuated Listeria monocytogenes strains augment expression of foreign immunogens and enhance vaccine-elicited humoral and cellular immune responses. Infect Immun.

[68] Lauer P, Chow MY, Loessner MJ, Portnoy DA, Calendar R (2002). Construction, characterization, and use of two Listeria monocytogenes site-specific phage integration vectors. J Bacteriol.

[69] Lyons CR (2004). Murine model of pulmonary anthrax: kinetics of dissemination, histopathology, and mouse strain susceptibility. Infect Immun.

