# Supplementary material for: Single vector platform vaccine protects against lethal respiratory challenge with Tier 1 select agents of anthrax, plague, and tularemia
Source: Sci Rep. 2018 May 3;8:7009. doi: 10.1038/s41598-018-24581-y (PMC5934503; doi:10.1038/s41598-018-24581-y)
Supplement: Supplementary file 1 — Supplementary Information [file 41598_2018_24581_MOESM1_ESM.docx]

**Single vector platform vaccine protects against lethal respiratory challenge with Tier 1 select agents of anthrax, plague, and tularemia**

Qingmei Jia^1^, Richard Bowen^2^, Barbara Jane Dillon^1^, Saša Masleša-Galić^1^, Brennan T. Chang^1^, Austin C. Kaidi^1^, and Marcus A. Horwitz^1,*^

^1^ Division of Infectious Diseases, Department of Medicine, 37-121 Center for Health Sciences, School of Medicine, University of California – Los Angeles, 10833 Le Conte Avenue, Los Angeles, CA 90095-1688

^2^ Department of Biomedical Sciences, Colorado State University, Fort Collins, CO 80523

^*^Corresponding author:

[MHorwitz@mednet.ucla.edu](mailto:mhorwitz@mednet.ucla.edu)

**Supporting Information**

**Supporting Methods**

**Immunization of mice for vaccine dissemination and clearance**. To assay dissemination and clearance of rLVS Δ*capB* vaccines, mice were immunized intradermally (i.d.) or intranasally (i.n.) with 10^6^ CFU each of LVS Δ*capB,* or rLVS Δ*capB*/*Ba*; or intramuscularly (i.m.) or (i.n.) with 10^6^ CFU each of Lm Δ*actA* Δ*inlB* *prfA* (Lm vector), rLm Δ*actA* Δ*inlB* *prfA*/LLOss-Ba (rLm/Ba), or rLm Δ*actA* Δ*inlB* *prfA*/LLOss-Yp (rLm/Yp) vaccines*.* The immunized mice were euthanized at days 1, 4, 7, 14, and 21 post-vaccination; and their spleen, lung, liver, draining lymph nodes, and local skin at i.d. injection site (at the base of tail) (i.d. route only) were removed and assayed for bacterial CFU ^1, 2^.

**Heterologous protein expression by and growth kinetics of LVS Δ*capB*- and Listeria-vectored vaccines in broth culture and in infected macrophage-like cells**

To assess protein expression by rLVS Δ*capB* vaccines in broth culture, we inoculated each of the vaccine stocks into 3% tryptic soy broth supplemented with 0.1% L-cysteine (TSBC), grew them overnight, collected bacterial cells, lysed the cells in SDS buffer, applied equivalent amounts of lysates to SDS-PAGE, and analyzed protein expression by Western blotting. Secreted proteins in the supernate of Brain Heart Infusion (BHI) broth culture of rLm vaccines were precipitated by the TCA-acetone method and analyzed by Western blotting. Monoclonal antibodies specific to *B. anthracis* PA (BEI, DD-9) and goat polyclonal antisera specific to *Y. pestis* LcrV (BEI, NR-31022) were used as primary antibody in Western blotting. Growth kinetics of rLVS Δ*capB* and rLm vaccines in broth and in infected macrophages were assayed as described previously ^1, 2, 3^. Specifically, monocytic THP-1 cells were seeded at 3x10^5^ cells/well on 24-well plates and differentiated in the presence of PMA for 3 days. Vaccine vector (LVS Δ*capB*) and rLVS Δ*capB* vaccines were grown on Chocolate agar supplemented without (vector) or with (vaccines) kanamycin (7.5 μg/ml) for 3 days. The differentiated THP-1 cells were left uninfected or infected with, rLVS Δ*capB*/Ba or rLVS Δ*capB*/Yp opsonized with human serum and incubated at 37°C for 1 h. The cells were then washed with RPMI three times and incubated with complete RPMI supplemented with gentamycin (0.1μg/ml) to inhibit extracellular bacterial growth. At 24 h post infection, medium was removed from wells; cells were lysed, and cell lysates analyzed by Western blotting using a mixture of monoclonal antibody to *B. anthracis* PA antigen and goat polyclonal antibody to *Y. pestis* LcrV antigen.

**Anthrax toxin neutralizing activity.** Sera were assayed for the titer of toxin-neutralizing activity (TNA) based on their ability to inhibit the cytotoxicity of anthrax toxin, the combination of PA and LF ^4^. Briefly, J774A.1 cells were harvested, seeded at ~ 3 x 10^4^ cells/well in 96-well microtiter plates, and incubated at 37°C in a 5% CO2 incubator for 17 – 19 h prior to the assay. A positive neutralization control serum [Anthrax Vaccine Adsorbed (AVA) pooled human plasma, BEI NR-28672], a negative control serum (normal pooled human plasma, BEI NR-28671), medium, and sera from vaccinated and control mice were prepared in separate 96-well microtiter plates. Mouse sera were serially diluted 2-fold seven times at a starting dilution of 1:20. The positive control was serially diluted 2-fold twelve times at a starting dilution of 1:100; and the negative control diluted 1:100. In triplicate, the serially diluted immune serum, positive control serum, negative control serum, and medium were incubated with anthrax lethal toxin [50 ng/ml PA (BEI NR-3780) and 40 ng/ml LF (BEI NR-4368)] at 37°C for 30 min. After the culture medium was removed from the J774A.1 cell monolayer, the toxin–serum and toxin-medium (toxin only control) mixes were transferred (100 μl/well) to the J774A.1 cell monolayer, and the incubation continued for 4 h. Cell viability was determined by addition of 20 µl AQ One solution (Promega) and incubation continued for 1 h. The assay was terminated by adding 25 μl of 10% SDS to each well. Optical density was read at 490 nm by a microplate reader (iMark, BioRad). The endpoint titer of TNA is expressed as the log_10_ value of the reciprocal of the last dilution yielding an OD that is greater than twice the background value of J774A.1 cells incubated with only lethal toxin ^5^.

**Supporting Tables and Figures**

**Table S1. Attenuated *F. tularensis* LVS Δ*capB* and *L. monocytogenes* vectored vaccines expressing immunoprotective antigens of Ft, Ba and Yp**

| Strain | Description | Reference |
| --- | --- | --- |
| LVS | Live vaccine strain | CDC |
| LVS Δ*capB* | Unmarked LVS with deletion of *capB* | ^6^ |
| rLVS Δ*capB*/iglABC | LVS Δ*capB* carrying shuttle plasmid pFNL/*pbfr-iglABC(GGSG)* | ^1^ |
| rLVS Δ*capB*/Ba | LVS Δ*capB* carrying shuttle plasmid pFNL/*p*bfr-BaLFnPAc(GGSG) | This study |
| rLVS Δ*capB/*Yp | LVS Δ*capB* carrying shuttle plasmid pFNL/*pomp*-YpF1V(GGSG) | This study |
| Lm Δ*actA* Δ*inlB* *prfA* | Lm Δ*actA* Δ*inlB* ΔuvrAB *prfA* (*G155S*) | ^7^ |
| rLm Δ*actA* Δ*inlB* *prfA*/  ActAN-Ba | rLm Δ*actA* Δ*inlB* *prfA* (*G155S*) with an ActAN-BaLFnPAc(GGSG) expression cassette integrated at tRNA^arg^ locus | This study |
| rLm Δ*actA* Δ*inlB* *prfA*/LLOss-Ba | rLm Δ*actA* Δ*inlB* *prfA* with an LLOss-BaLFnPAc(GGSG) expression cassette integrated at tRNA^arg^ locus | This study |
| rLm Δ*actA* Δ*inlB* *prfA*/  ActAN-Yp | rLm Δ*actA* Δ*inlB* *prfA* with an ActAN-YpF1V(GGSG) expression cassette integrated at tRNA^arg^ locus | This study |
| rLm Δ*actA* Δ*inlB* *prfA*/  LLOss-Yp | rLm Δ*actA* Δ*inlB* *prfA* with an LLOss-YpF1V(GGSG) expression cassette integrated at tRNA^arg^ locus | This study |

Fig. S1


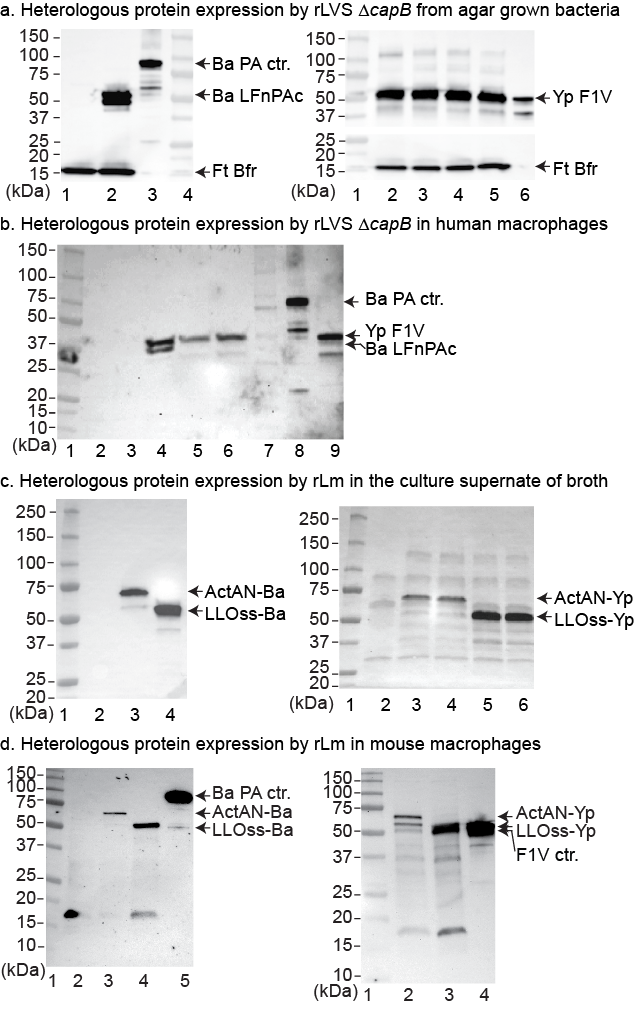


**Fig. S1. Expression of heterologous fusion proteins of *B. anthracis* and *Y. pestis* by rLVS** Δ***capB* and rLm Δ*actA* Δ*inlB prfA* vaccines in broth and in infected macrophage-like cells*.*** **a.** Expression of *B. anthracis* (left) and *Y. pestis* (right) fusion proteins by rLVS Δ*capB* grown on an agar plate. Single colonies of chocolate agar grown rLVS Δ*capB/*Ba and rLVS Δ*capB/*Yp (4 clones) were lysed in SDS sample buffer, and the lysates analyzed by Western blotting using a mixture of antibody to *B. anthracis* PA and antibody to *F. tularensis* Bfr (left panel) or antibody to *Y. pestis* LcrV protein followed by antibody to Bfr (right panel). Left panel, lane 1, LVS Δ*capB* vector; lane 2, rLVS Δ*capB/*Ba; lane 3, PA protein control (PA ctr.); lane 4, protein mass standards. Right panel, lane 1, protein mass standards; lanes 2-5, rLVS Δ*capB/*Yp; lane 6, monomer of F1V protein control. **b.** Expression of fusion proteins by rLVS Δ*capB* in infected human macrophage-like cells. Monocytic THP-1 cells seeded on 24-well plates and differentiated in the presence of PMA were left uninfected or infected with LVS Δ*capB*, rLVS Δ*capB*/Ba or rLVS Δ*capB*/Yp; cells were lysed at 24 h post infection, and cell lysates analyzed by Western blotting using a mixture of antibody to *B. anthracis* PA and to *Y. pestis* LcrV. Lanes 1 & 7, two different protein standards; lane 2, uninfected control; lane 3, LVS Δ*capB;* lane 4, rLVS Δ*capB*/Ba; lanes 5 and 6, two clones of rLVS Δ*capB/*Yp vaccines; lane 8, *B. anthracis* PA and degraded proteins; lane 9, *Y. pestis* F1V monomer protein and degraded proteins. **c**. Expression and secretion of heterologous fusion proteins by rLm vaccines in broth. Culture filtrates of Lm vector or rLm vaccines were analyzed by Western blotting using antibody to *B. anthracis* PA (left panel) or to *Y. pestis* LcrV (right panel). Left panel, lane 1, protein mass standards; lane 2, Lm vector; lane 3, rLm/ActAN-Ba; lane 4, rLm/LLOss-Ba. Right panel, lane 1, protein mass standards; lane 2, Lm vector; lanes 3 & 4, two clones of rLm/ActAN-Yp; lanes 5 & 6, two clones of rLm/LLOss-Yp. **d**. Expression of heterologous fusion proteins by rLm vaccines in infected mouse macrophage-like cells. Monolayers of J774 cells were not infected or infected with a stationary culture of rLm vaccines similarly as described above in the legend to c. Lysates were subjected to Western blotting analysis using antibody to *B. anthracis* PA (left) or to *Y. pestis* LcrV (right). Left panel, lane 1, protein standards; lane 2, uninfected control; lane 3, rLm*/*ActAN-Ba; lane 4, rLm*/*LLOss-Ba; lane 5, PA protein. Right panel, lane 1, protein standards; lane 2, rLm*/*ActAN-Yp; lane 3, rLm*/*LLOss-Yp; lane 4, F1V protein control (F1V ctr.). **a - d**. On the left border of each panel are listed the masses of protein standards; on the right border are listed the proteins of interest. Each blot was processed by using the Bio-Rad imaging system (ChemiDoc XRS) and Quantity One software, which allows the overlap of a white-light image, for visualization of the protein standards (a, left panel lane 4 and right panel lane 1; b - d, lane 1), and a chemiluminescent image, for visualization of the antibody-labeled protein bands.

Fig. S2


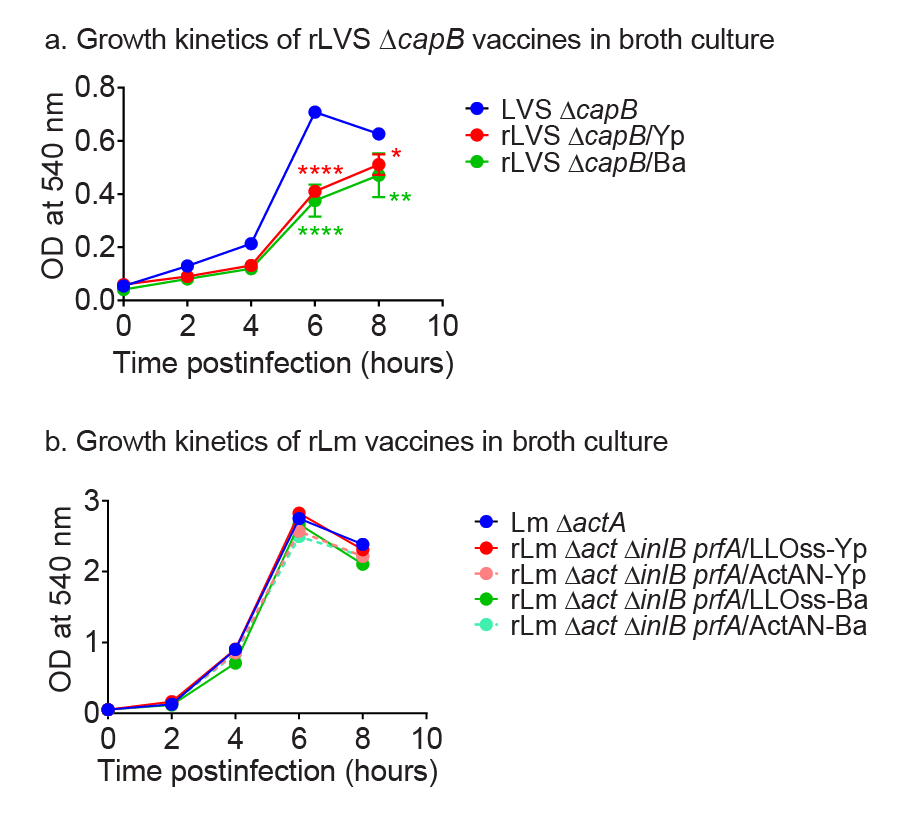


**Fig. S2. Growth kinetics of LVS Δ*capB*- and Lm-vectored vaccines in broth culture**. **a**. LVS Δ*capB*-vectored vaccines. To follow the growth kinetics of an individual vaccine strain in broth, we cultured LVS Δ*capB*-vectored vaccines on chocolate agar for 3 days, scraped the colonies into Chamberlain Defined Medium (CDM) ^8^, and grew them in CDM overnight in the presence of antibiotic selection. The overnight culture was subsequently diluted in CDM in the presence of antibiotic selection, adjusted to an optical density of 0.1 at 540 nm, and incubated at 37⁰C with vigorous shaking. At 0, 2, 4, 6, and 8 hours post inoculation, we removed one aliquot of each individual strain aseptically and measured the optical density at 540 nm. Shown are growth curves representative of three similar experiments. *, *P* < 0.05; **, *P* < 0.01; ****, *P* *<* 0.0001 vs. LVS Δ*capB* vector group by Two-way ANOVA with Tukey’s post comparisons test (Prism). **b.** Lm-vectored vaccines. We inoculated the glycerol stock of each rLm vaccine in Brain Heart Infusion (BHI) broth and cultured them overnight at 37⁰C without shaking. The overnight culture was subsequently diluted in BHI in the presence of antibiotic selection, adjusted to an optical density of 0.05 at 540 nm, and incubated at 37⁰C with vigorous shaking. At 0, 2, 4, 6, and 8 hours post inoculation, we removed one aliquot of each individual strain aseptically and measured the optical density at 540 nm. Shown are growth curves representative of two similar experiments. There was no significant difference in growth kinetics between the Lm vector and any of the rLm vaccines.

Fig. S3

**
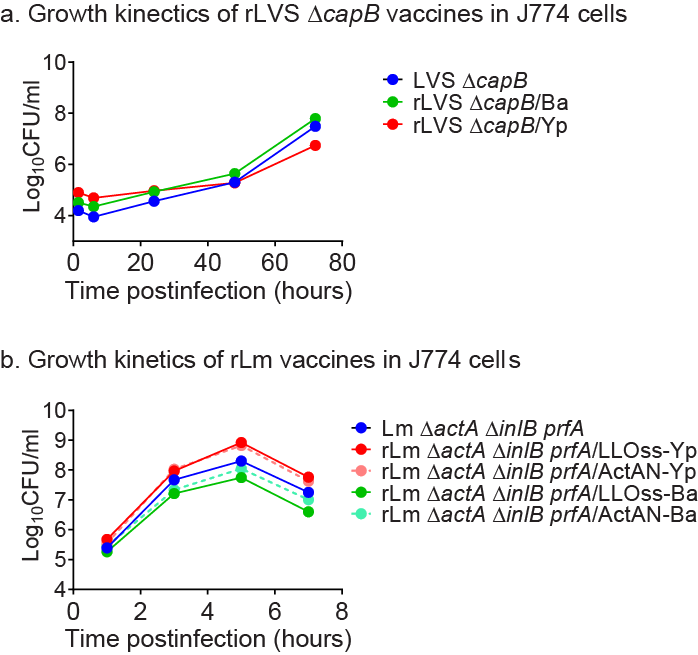
**

**Fig. S3. Growth kinetics of rLVS Δ*capB* and rLm vaccines in mouse macrophage-like J774 cells. a**. rLVS Δ*capB*-vectored vaccines. To follow the growth kinetics of an individual vaccine strain in mouse macrophage-like cells, we cultured LVS Δ*capB*-vectored vaccines on chocolate agar for 3 days, scraped the colonies into Chamberlain Defined Medium (CDM) ^8^, and grew them in CDM overnight in the presence of antibiotic selection. Monolayers of J774 cells were infected with the vaccine vector or rLVS Δ*capB* vaccines opsonized with human serum and incubated at 37°C for 1.5 h. The cells were then washed with DMEM three times and incubated with complete DMEM supplemented with gentamycin (0.1μg/ml) to inhibit extracellular bacterial growth. At various times post infection, medium was removed from wells and the cells were lysed in 0.1% Saponin in PBS buffer. The cell lysates were serially diluted, plated on Chocolate agar supplemented with (vaccines) or without (vector) kanamycin, incubated for 3 – 4 days at 37⁰C, and colonies counted. **b.** rLm-vectored vaccines. We inoculated the glycerol stock of each rLm vaccine in Brain Heart Infusion (BHI) broth and cultured overnight at 30⁰C without shaking. Monolayers of J774 cells were infected with the vaccine vector or vaccines opsonized with human serum and incubated at 37°C for 1.0 h. The cells were then washed with DMEM three times and incubated with complete DMEM supplemented with gentamycin (10 μg/ml) to inhibit extracellular bacterial growth. At various times post infection, medium was removed from wells and cells were lysed in 0.1% Saponin in PBS buffer. The cell lysates were serially diluted and plated on BHI agar supplemented with streptomycin (vector) or streptomycin and erythromycin (vaccines); the agar plates were incubated overnight at 37⁰C; and colonies counted. Shown are results representative of two (rLm) or more (rLVS Δ*capB*) experiments.

Fig. S4


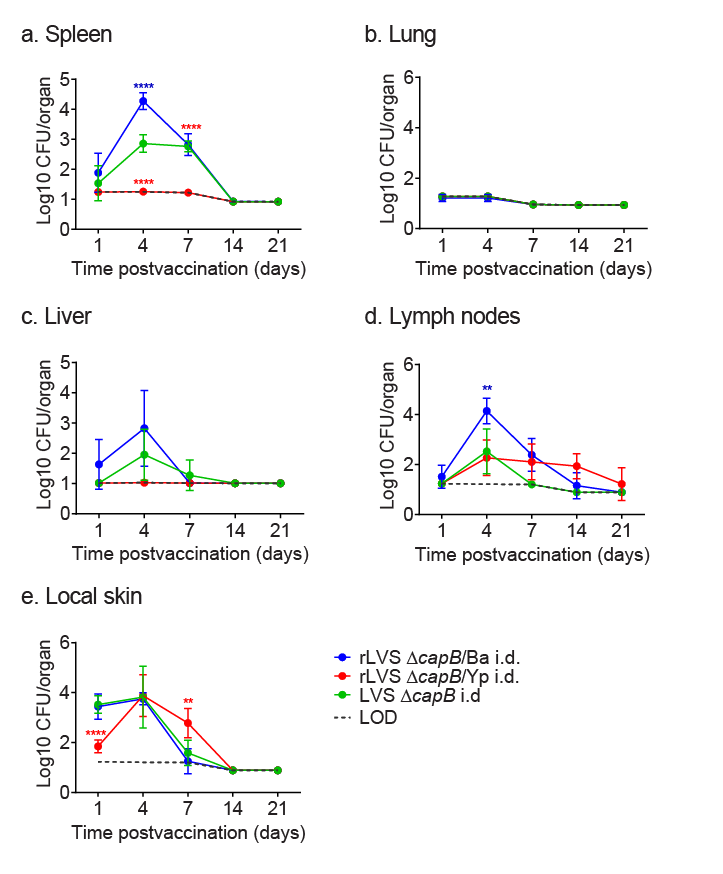


**Fig. S4. Clearance of rLVS Δ*capB*-vectored *B. anthracis* and *Y. pestis* vaccines after systemic vaccination.** Mice were immunized intradermally (i.d.) with rLVS Δ*capB*/Ba, rLVS Δ*capB*/Yp, or the LVS Δ*capB* vector (10^6^ CFU of each vaccine) at Day 0; euthanized (4 mice/group/time point) at Day 1, 4, 7, 14 and 21; and their spleens (a), lungs (b), livers (c), draining lymph nodes (inguinal) (d) and skin injection sites (local skin) (e) removed and assayed for bacterial burden. Values are mean ± SEM of log_10_ CFU per organ for n = 4 per group per time point. Differences in log_10_ CFU among individual groups were compared by two-way ANOVA with Tukey’s multiple comparisons test. Values that are significantly different from that for the LVS Δ*capB* vector control at each time point are marked with asterisk(s) color-coded to the vaccine symbols. **, *P* < 0.01; ****, *P* < 0.0001. LOD, Limit of detection. After i.d. vaccination, rLVS Δ*capB*/Ba disseminated to the spleen and draining lymph nodes faster and grew to a significantly higher level than the parental LVS Δ*capB* strain at the peak of infection (Day 4 post vaccination); in contrast, rLVS Δ*capB*/Yp showed delayed replication at the vaccination site (local skin) and significantly delayed systemic dissemination. All the vaccines were cleared by day 21 post vaccination.

Fig. S5

**
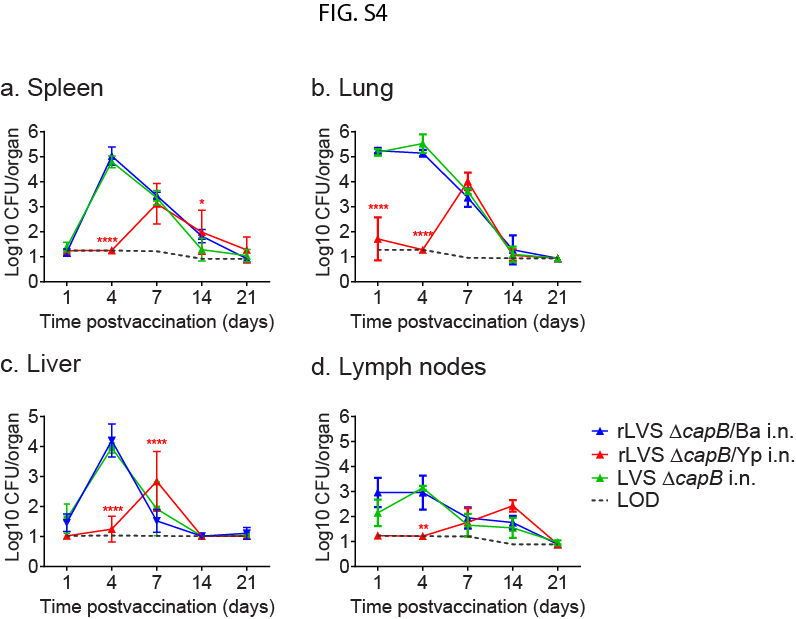
**

**Fig. S5. Clearance of rLVS Δ*capB*-vectored *B. anthracis* and *Y. pestis* vaccines after mucosal vaccination.** Mice were immunized intranasally (i.n.) with rLVS Δ*capB*/Ba, rLVS Δ*capB*/Yp, or the LVS Δ*capB* vector (10^6^ CFU of each) at Day 0; euthanized (4 mice/group/time point) at Day 1, 4, 7, 14 and 21; and their spleens (a), lungs (b), livers (c), and draining lymph nodes (tracheobronchial) (d) removed and assayed for bacterial burden. Values are mean ± SEM of log_10_ CFU per organ for n = 4 per group per time point. Differences in log_10_ CFU among individual groups were compared by two-way ANOVA with Tukey’s multiple comparisons test. Values that are significantly different from that for the LVS Δ*capB* vector control at each time point are marked with asterisk(s) color-coded to the vaccine symbols. **, *P* < 0.01; ****, *P* < 0.0001. LOD, Limit of detection.

Fig. S6

**
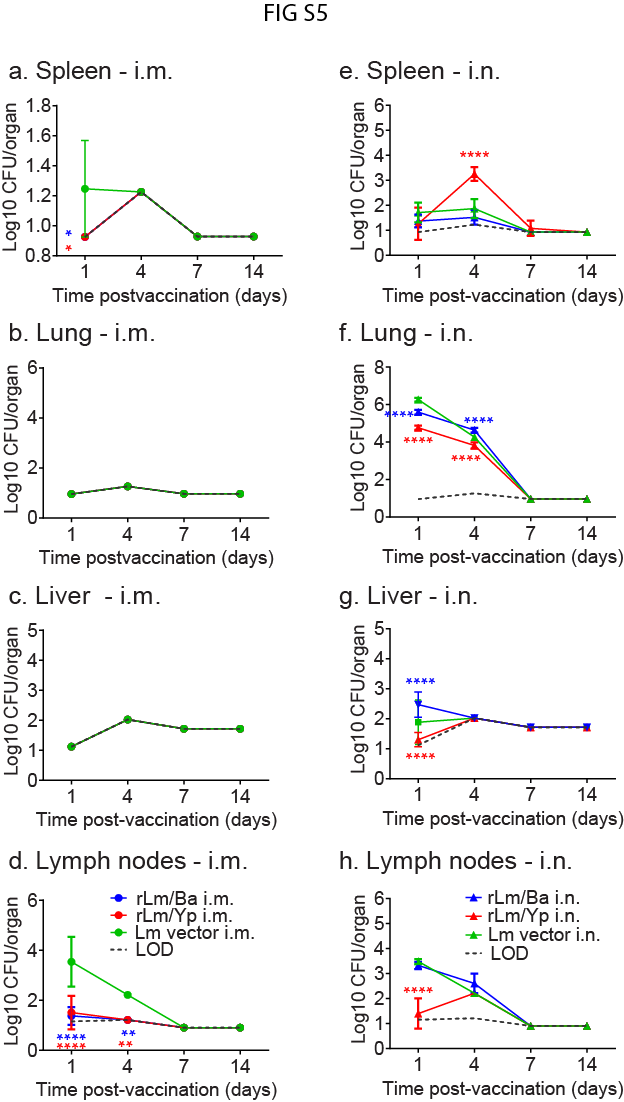
**

**Fig. S6. Clearance of rLm-vectored *B. anthracis* and *Y. pestis* vaccines after systemic and mucosal vaccination.** Mice were immunized intramuscularly (i.m.) or intranasally (i.n.) with rLm/Ba, rLm/Yp, or the Lm vector (Lm Δ*actA* Δ*inlB prfA*) (10^6^ CFU of each vaccine) at Day 0; euthanized (4 mice/group/time point) at Day 1, 4, 7, and 14; and their spleens (a, e), lungs (b, f), livers (c, g), and draining lymph nodes (inguinal for i.m. and tracheobronchial for i.n.) (d, h) removed and assayed for bacterial burden. Values are mean ± SEM of log_10_ CFU per organ for n = 4 per group per time point. Differences in log_10_ CFU among individual groups were compared by two-way ANOVA with Tukey’s multiple comparisons test. Values that are significantly different from that for the LVS Δ*capB* vector control at each time point are marked with asterisk(s) color-coded to the vaccine symbols. **, *P* < 0.01; ****, *P* < 0.0001. LOD, Limit of detection.

Fig. S7

**
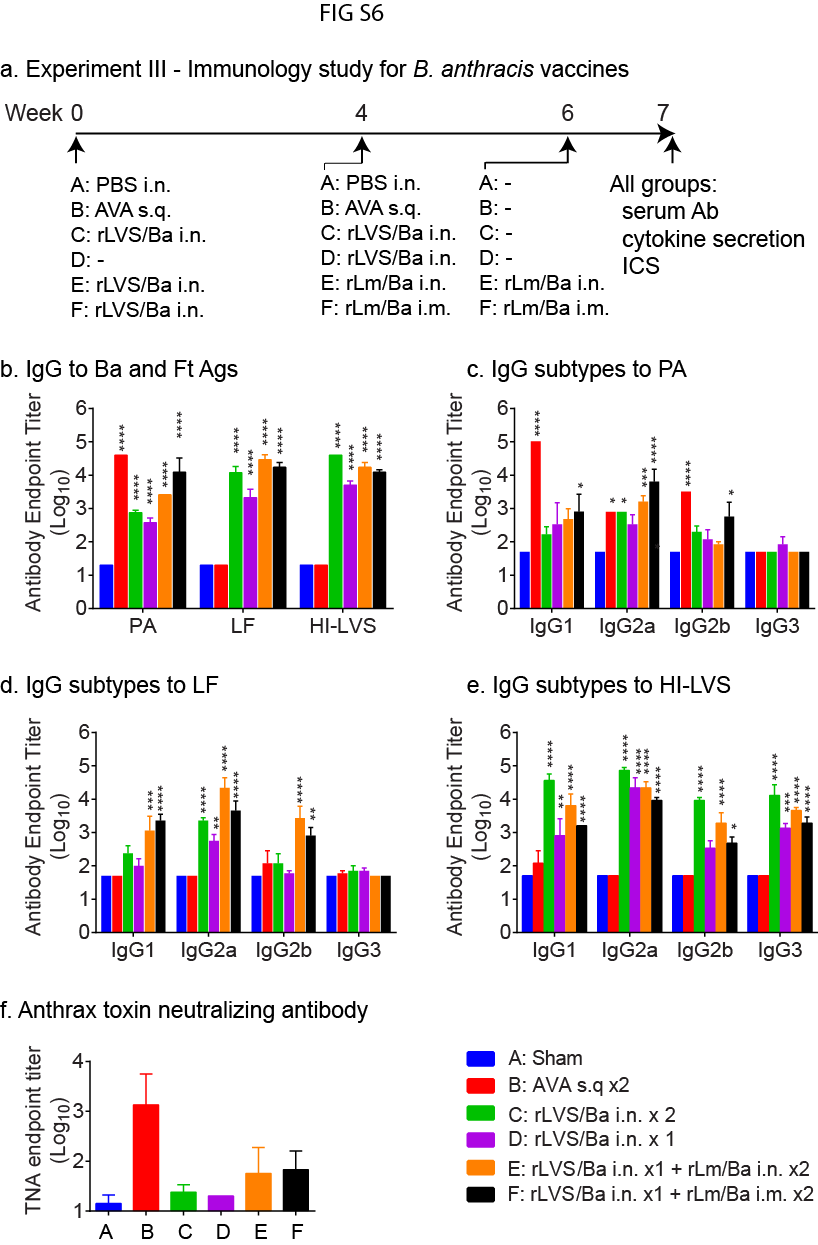
**

**Fig. S7. Antigen-specific humoral immune responses induced by homologous prime-boost vaccination with rLVS Δ*capB*/Ba or heterologous prime-boost vaccination with rLVS Δ*capB*/Ba - rLm Δ*actA* Δ*inlB* *prfA*/Ba. a.** Experiment schedule (the same experiment as Experiment III shown in Fig. 4 and 5). For homologous prime-boost vaccination, mice (n = 4/group) were immunized with rLVS Δ*capB*/Ba (rLVS/Ba) (10^6^ CFU) intranasally (i.n.) twice at weeks 0 and 4 or once at Week 4. For heterologous prime-boost vaccination, mice were primed with rLVS Δ*capB*/Ba i.n. at Week 0 and boosted with rLm Δ*actA* Δ*inlB prfA*/Ba (rLm/Ba) i.n. or intramuscularly (i.m.) twice at Weeks 4 and 6, as indicated. Control mice were immunized with PBS i.n. or AVA subcutaneously (s.q.) twice at Weeks 0 and 4. At week 7, all mice were bled; euthanized; their sera assayed for antibody; and their lungs and spleens processed for assays of cytokine secretion and intracellular cytokine staining (ICS). **b.** IgG to *B. anthracis* and *F. tularensis* antigens. Sera were assayed for IgG specific to *B. anthracis* Protective Antigen (PA), lethal factor (LF) and heat-inactivated *F. tularensis* Live Vaccine Strain (HI-LVS), as indicated. **c – e**. IgG subtypes to PA, LF and HI-LVS. Sera were assayed for IgG subtypes IgG1, IgG2a, IgG2b, and IgG3 to PA (c), LF (d) and HI-LVS (e) as indicated. **f**. Anthrax toxin neutralizing antibody. Sera were assayed for toxin neutralizing antibody endpoint titer by preincubating serum dilutions with Lethal Toxin (PA 50 ng/ml with LF 40 ng/ml) before adding the mixture to J774 macrophage-like cells and subsequently incubating with AQueous One solution (Promega); the endpoint titer is expressed as the highest serum dilution yielding an optical density at 490 nm greater than twice the background level of macrophage-like J774A.1 cells incubated with only Lethal toxin (Boyaka et al, J. Immunol, 2003). Values are mean + SD. ***P* < 0.01; ***, *P* < 0.001, ****, *P* < 0.0001 by two-way ANOVA with Tukey’s post comparisons test (Prism).

Fig. S8**
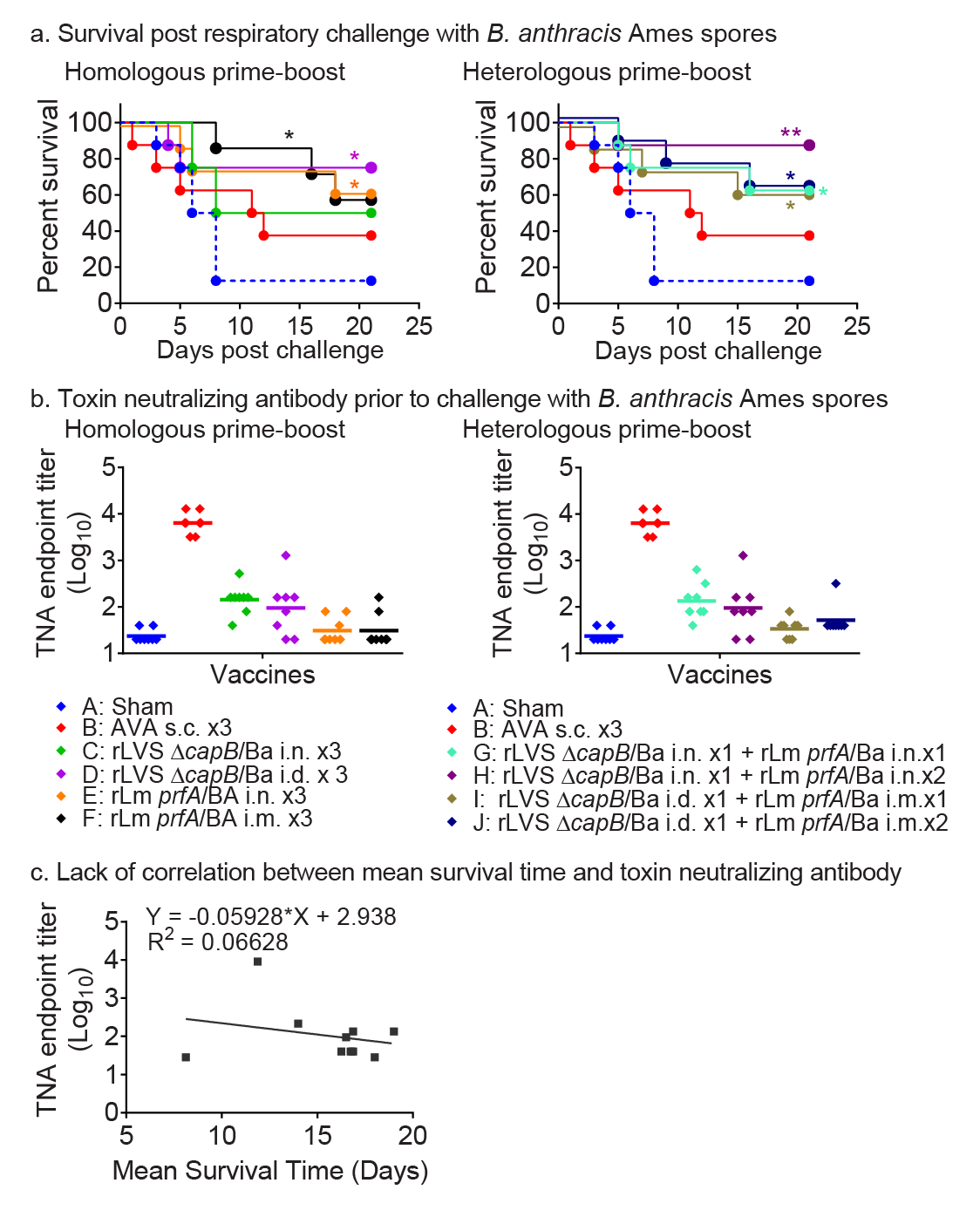
**

**Fig. S8.**  **Three immunizations by homologous prime-boost vaccination with rLVS Δ*capB*/Ba or by heterologous prime-boost vaccination with rLVS Δ*capB*/Ba – rLm Δ*actA* Δ*inlB prfA*/Ba induce anthrax toxin neutralizing antibody that is not correlated with protective immunity.** BALB/c mice, 8/group, were immunized, bled one week prior to i.n. challenge with *B. anthracis* Ames spores, challenged, and monitored for 3 weeks post challenge, as indicated in Fig. 3a. **a**. Survival post challenge. Survival curves between control and vaccinated mice were compared by log-rank test (Mantel-cox, Prism); *P* values that are significantly different from the Sham group are color-coded to the color of the vaccine symbol. *, *P* < 0.05 and **, *P* < 0.01. (as also shown in Fig. 3b, bottom panels). **b**. TNA prior to challenge. Toxin neutralizing antibody (TNA) titers were quantitated by the capacity of serial dilutions of immunized mouse sera to protect murine macrophages (J774A.1) from anthrax lethal toxin (PA and LF), as described above in Supporting Materials. The TNA endpoint titer is expressed as the log_10_ value of the reciprocal of the highest dilution yielding an OD (viability measurement) that is greater than twice the background value of J774A.1 cells incubated with lethal toxin in the absence of immune sera. **c**. Lack of correlation between pre-challenge serum TNA endpoint titer and mean survival time. A linear regression was used to obtain values for slope and intercept and the correlation coefficient (R^2^) between pre-challenge toxin neutralizing activity (TNA) and mean survival time (days) at 21 days post challenge was calculated (R^2^ =0.07). In addition, a Two-tailed *P* value was calculated that showed that TNA and mean survival time were not significantly correlated (*P* = 0.47).

Fig. S9


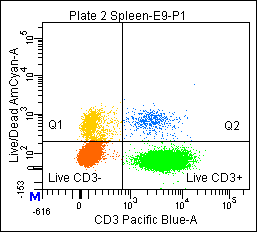

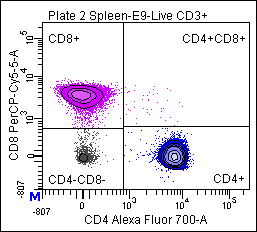

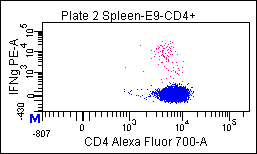

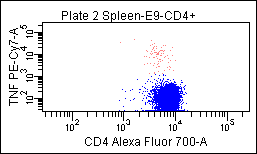

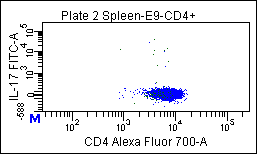

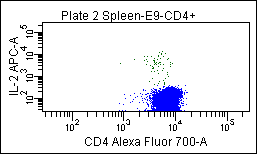

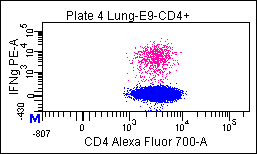

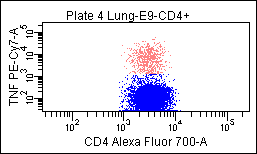

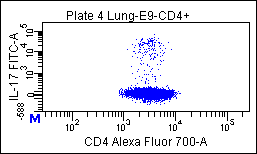

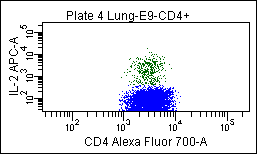


IFN-γ

IL-2

TNF-α

IL-17

Lung - CD8+

Spleen - CD4+

Lung - CD4+

51%

34%

3.9%

0.2%

61.9%

0.66%

0.48%

0.68%

0.07%

11.3%

4.9%

10.4%

3.1%


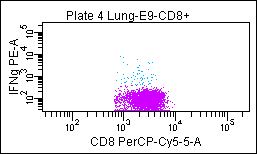

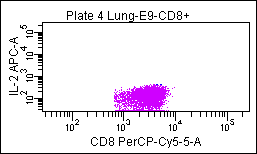

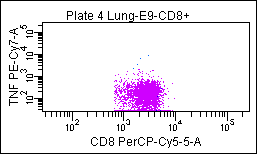

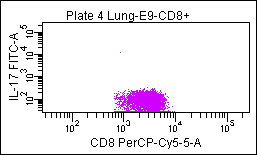


0.26%

0%

0.03%

0.01%

a. Gating of live CD3+ cells

b. Gating of CD4+ vs. CD8+

c. Cytokine-producing CD4+ and CD8+ T cells

**Fig. S9. Representative plots of cytokine-producing CD4+ and CD8+ T cell populations in the spleen and lung after immunization with *B. anthracis* vaccines.** As indicated in Fig. 4a, mice were primed with rLVS Δ*capB*/Ba i.n at Week 0 and boosted with the heterologous rLm Δ*actA* Δ*inlB* *prfA*/Ba vaccine i.n. (Group E) twice at Weeks 4 and 6. At Week 7, mice were euthanized; their lungs and spleens removed; and single cell suspensions of lung and spleen cells prepared and stimulated with LF protein (2 µg/ml) for 6 hours and then stained for cell surface and intracellular markers. A minimum of 50,000 events were acquired in HT LSRII (BD Biosciences) and analyzed by FACS DIVA (BD Biosciences) for the frequency of live CD3+CD4+ or CD3+CD8+ T cells expressing single or multiple cytokines among IFN-γ, IL-2, TNF-α, and IL-17. Shown are the plots from one mouse. **a**. Gating of live splenic CD3+ T cells. The frequency of live CD3+ T cells is shown in the lower right quadrant. **b.** Gating of live splenic CD3+CD8+ and CD3+CD4+ T cells. Live CD3+ T cells from panel (a) were gated for CD4+, CD8+, double negative and double positive T cells (the frequency of each of the subtypes is shown within each plot). **c**. Frequencies of cytokine-producing lung CD8+ T cells or spleen CD4+ and CD8+ T cells. At the top of the panels are shown the cell populations analyzed; to the right of the panels are the cytokines analyzed; and within each plot are the frequencies of ­­antigen-stimulated lung CD8+ or spleen CD4+ or CD8+ T cells expressing IFN-γ, IL-2, TNF-α, or IL-17. Pseudo colors were used to differentiate various cell populations.

Fig. S10


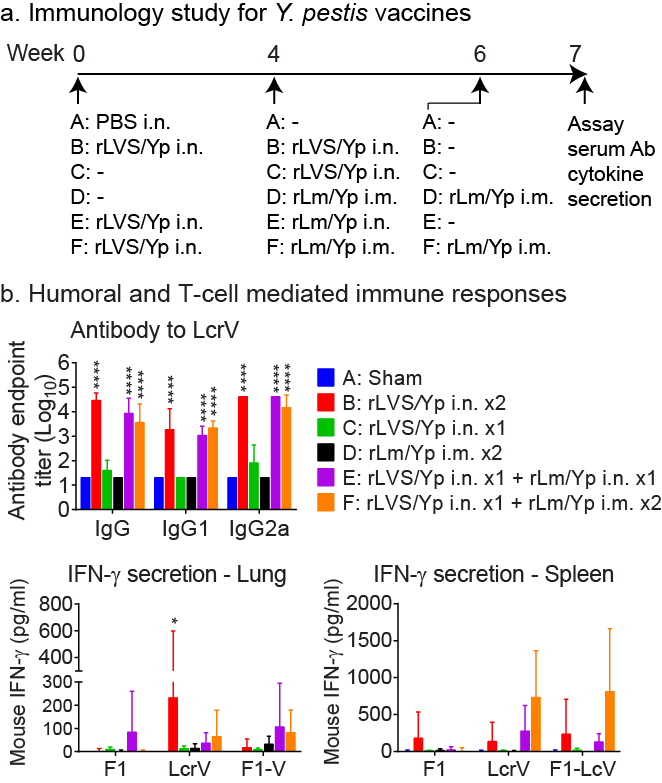


**Fig. S10. Antigen specific humoral or cell-mediated immune responses induced by homologous prime-boost vaccination with rLVS Δ*capB*/Yp or rLm Δ*actA* Δ*inlB* *prfA*/Yp, or heterologous prime-boost vaccination with LVS Δ*capB*/Yp - Lm Δ*actA* Δ*inlB prfA*/Yp. a.** Experiment schedule. Mice were immunized homologously with rLVS Δ*capB*/Yp (rLVS/Yp) i.n. or rLm/Yp i.m. or heterologously by priming with rLVS/Yp i.n. and subsequently boosting with rLm/Yp i.n. or i.m. at Weeks 0, 4 and 6, as indicated. Control mice were sham-immunized with PBS. At Week 7, all mice were bled and spleens and lungs removed. **b.** Humoral and T-cell mediated immune responses. Top panel: sera were assayed for IgG and subtypes IgG1 and IgG2a to *Y.* *pestis* antigen F1-LcrV monomer protein. The endpoint titer of serum antibody is defined as the mean log dilution that yields an OD greater than the mean OD of Sham sera plus three standard deviations at the same serum dilution. Bottom panels**:** Single cell suspensions of lung (left) and spleen (right) cells were stimulated with medium alone or medium supplemented with F1, LcrV, or F1-LcrV monomer proteins, and their supernatants assayed for IFN-γ. Values shown are the mean + SEM after subtraction of values for medium alone. *, *P* < 0.05 and ****, *P* < 0.0001 by two-way ANOVA with Tukey’s multiple comparisons test (Prism).

References

1. Jia Q, Bowen R, Lee BY, Dillon BJ, Maslesa-Galic S, Horwitz MA. Francisella tularensis Live Vaccine Strain deficient in capB and overexpressing the fusion protein of IglA, IglB, and IglC from the bfr promoter induces improved protection against F. tularensis respiratory challenge. *Vaccine* **34**, 4969-4978 (2016).

2. Jia Q, Dillon BJ, Maslesa-Galic S, Horwitz MA. Listeria-vectored vaccine expressing the Mycobacterium tuberculosis 30 kDa major secretory protein via the constitutively active prfA* regulon boosts BCG efficacy against tuberculosis. *Infect Immun* **85**, e00245-00217 (2017).

3. Jia Q, Lee BY, Clemens DL, Bowen RA, Horwitz MA. Recombinant attenuated Listeria monocytogenes vaccine expressing Francisella tularensis IglC induces protection in mice against aerosolized Type A F. tularensis. *Vaccine* **27**, 1216-1229 (2009).

4. Li H*, et al.* Standardized, mathematical model-based and validated in vitro analysis of anthrax lethal toxin neutralization. *J Immunol Methods* **333**, 89-106 (2008).

5. Boyaka PN, Tafaro A, Fischer R, Leppla SH, Fujihashi K, McGhee JR. Effective mucosal immunity to anthrax: neutralizing antibodies and Th cell responses following nasal immunization with protective antigen. *J Immunol* **170**, 5636-5643 (2003).

6. Jia Q, Lee BY, Bowen R, Dillon BJ, Som SM, Horwitz MA. A Francisella tularensis live vaccine strain (LVS) mutant with a deletion in capB, encoding a putative capsular biosynthesis protein, is significantly more attenuated than LVS yet induces potent protective immunity in mice against F. tularensis challenge. *Infect Immun* **78**, 4341-4355 (2010).

7. Lauer P*, et al.* Constitutive Activation of the PrfA regulon enhances the potency of vaccines based on live-attenuated and killed but metabolically active Listeria monocytogenes strains. *Infect Immun* **76**, 3742-3753 (2008).

8. Chamberlain RE. Evaluation of Live Tularemia Vaccine Prepared in a Chemically Defined Medium. *Appl Microbiol* **13**, 232-235 (1965).
